# New light on plant ash glass found in Africa: Evidence for Indian Ocean Silk Road trade using major, minor, trace element and lead isotope analysis of glass from the 15^th^—16^th^ century AD from Malindi and Mambrui, Kenya

**DOI:** 10.1371/journal.pone.0237612

**Published:** 2020-08-13

**Authors:** Ieong Siu, Julian Henderson, Dashu Qin, Yu Ding, Jianfeng Cui, Hongjiao Ma

**Affiliations:** 1 School of Archaeology and Museology, Peking University, Beijing, China; 2 Department of Classics and Archaeology, University of Nottingham, University Park, Nottingham, United Kingdom; 3 University of Nottingham, Ningbo, China; University at Buffalo - The State University of New York, UNITED STATES

## Abstract

Seventeen glass vessels and twenty glass beads recovered from the excavations at the ancient city of Malindi and the archaeological site of Mambrui in Kenya, east Africa were analysed using electron probe microanalysis (EPMA) and laser ablation-inductively coupled plasma-mass spectrometry (LA-ICP-MS). The results show that all of the glass samples are soda-lime-silica glass. They belong to the high alumina -plant ash glass type, characterised by high alumina and relatively low calcium contents, widely distributed in eastern (10^th^– 16^th^ centuries AD) and southern Africa (13^th^ - 15^th^ centuries AD), Central Asia (9^th^– 14^th^ centuries AD) and southeast Asia (12^th^– 13^th^ centuries AD), made with plant ashes and sands. This is an understudied glass type for which previous research has indicated there were three types. When compared with published research on such glasses using Zr, Ti, Ba, Cr, La, Li, Cs, Na_2_O, MgO and CaO we have identified at least four different compositional groups of v-Na-Al glass: Types A, B, C and D. By comparing the results with contemporary v-Na-Al glass vessels and beads from Central Asia, Africa, and southeast Asia we show that most of the Malindi and Mambrui glass share similar characteristics to the compositions of Mapungubwe Oblate and some of the Madagascar glass beads from southern Africa. They belong to Type A v-Na-Al glass which is characterised by an elevated level of Ti and Ba and a relatively high ratios of Cr/La, relatively low Zr concentrations and low ratios of Zr/Ti. Differences in Zr, Li, MgO and Na_2_O concentrations in Type A glass indicates that there are subgroups which might derive from different glass workshop(s) specialising in Type A v-Na-Al glass production. Comparison with the chemical compositions of glass from Ghazni, Afghanistan and Termez, Uzbekistan, and by using lead isotope analysis, we suggest v-Na-Al glass was manufactured in Central Asia and possibly worked into vessels and beads there.

## Introduction

In recent years, large scale studies of African glass artefacts have been conducted using scientific methods such as LA-ICP-MS. These include the study of glass beads from southern Africa and east Africa, the studies by Marilee Wood and her colleagues of glass beads from Chibuene, southern Mozambique and from Zanzibar [1, 2] and the monumental study of southern African glass beads by Peter Robertshaw and his colleagues [3, 4]. Dussubieux and her colleagues [5, 6] published reviews of mineral soda alumina glass found in Africa and east Asia. Two types of glass were identified which circulated in Africa: (1) mineral soda alumina (designated m-Na-Al) glass and (2) plant ash alumina glass (designated v-Na-Al).

There is a general consensus that there are at least 5 m-Na-Al glass groups, based on the concentrations of U, Ba, Sr, Zr and Cs, circulating in eastern and southern Africa in the 9^th^– 18^th^ centuries AD [6]. It has been suggested that they were manufactured in India or in Sri Lanka. The low magnesia levels (< 1.5%) in m-Na-Al glass suggests that a mineral alkaline flux (e.g. *reh*) was used to make the glass, and the high alumina contents (c. 5% to 15%) suggests the use of a low quality sand [6].

**Fig 1:**
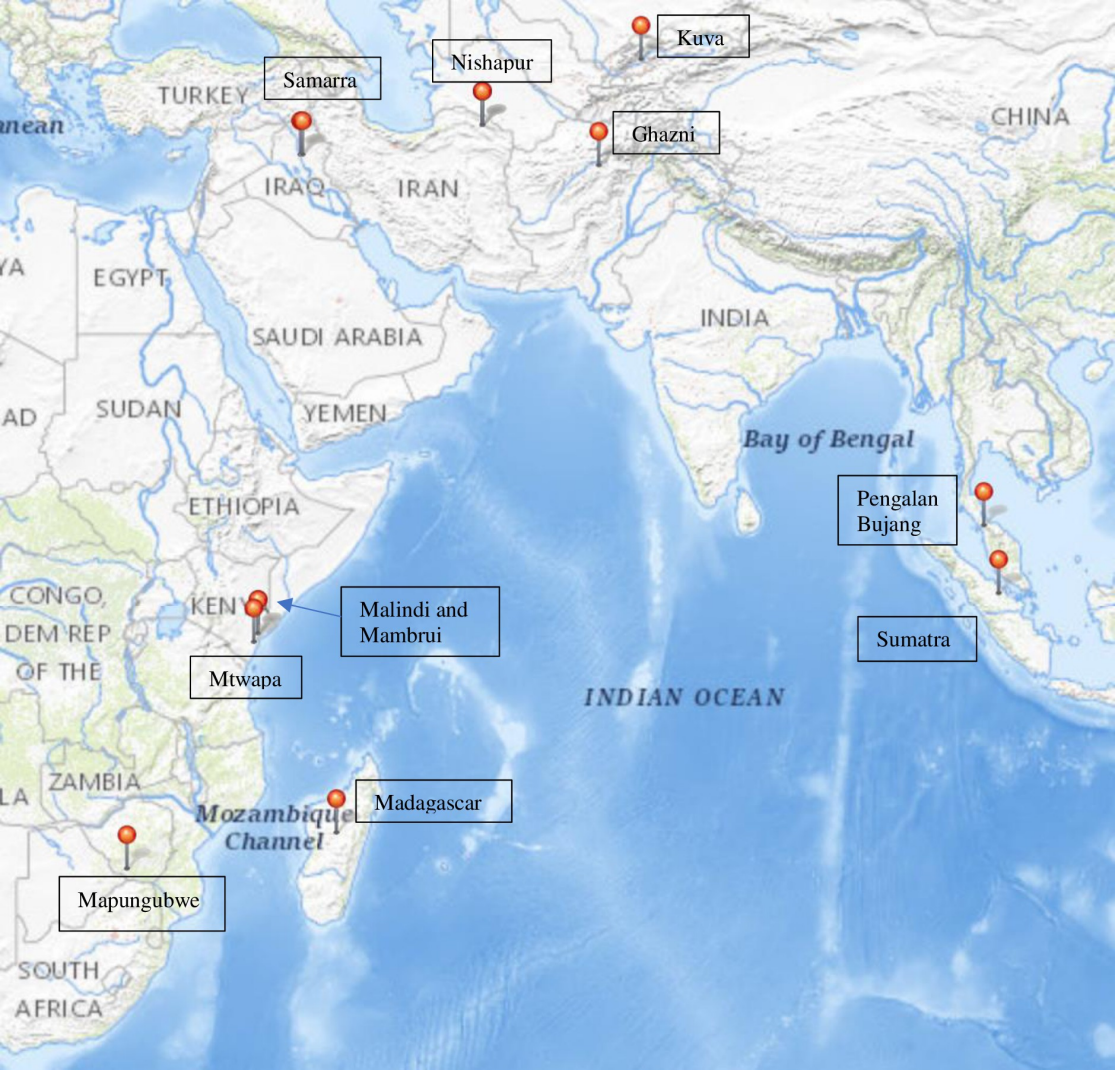
Locations of sites where v-Na-Al glass and low alumina soda plant ash glass have been found. V-Na-Al glass is found in: (1) Central Asia (9^th^– 14^th^ centuries AD)—Kuva and Aksiket in Uzbekistan, Ghazni in Afghanistan; (2) Kenya—Malindi, Mambrui (15^th^– 16^th^ centuries AD) and Mtwapa (10^th^– 17^th^ centuries AD); (3) Madagascar (13^th^– 14^th^ centuries AD); (4) southern Africa (13^th^– 15^th^ centuries AD)—Mapungubwe, south Africa; (5) southeast Asia—Pengalan Bujang in Malaysia (12^th^– 13^th^ centuries) and Sumatra in Indonesia (12^th^– 14^th^ centuries AD). Low alumina soda glass is found in Nishapur in Iran (9^th^– 10^th^ century AD) and Samarra (9^th^– 10^th^ century AD) in Iraq. (Photograph) courtesy of the U.S. Geological Survey: https://viewer.nationalmap.gov/advanced-viewer/viewer/index.html?extent=-6583299.154%2C-4681356.4382%2C19578955.3912%2C6178816.5406%2C102100.

The other type of high alumina soda glass, v-Na-Al, was made with plant ashes. This glass was also found in the Indian Ocean region and in southeast Asia during the 9^th^– 16^th^ centuries AD. High alumina plant ash glass was also found in Xinjiang, China but mainly dated to before the 4^th^ century AD [7]. Dussubieux *et al*. [6] and Robertshaw *et al*. [3, 4] have identified three types of v-Na-Al glass circulating in the Indian Ocean region. The first two types were found in beads from Mapungubwe, south Africa, Great Zimbabwe in Zimbabwe and Bosutwe in Botswana. The third glass type was used to make vessels. It has been found on the island of Sumatra in Indonesia, Pengalan Bujang in Malaysia and Mtwapa in Kenya (Fig 1). A limited amount of research has therefore been carried out on v-Na-Al glass and it is the least understood type of glass in the Indian Ocean region. Moreover, it is still not certain where the three different types of v-Na-Al glasses identified so far were made.

Thus, while chemical analyses have provided evidence for different types of African glass [4–6, 8, 9], we believe, by including our new data, it is possible to refine the identification of African glass types by examining trace elements associated with silica (Zr, Ti, La and Cr) and alkalies (Cs and Li). This approach has recently been used by Henderson *et al*. [10] and Shortland *et al*. [11] to provide a provenance and distinguish between production zones for Middle Eastern plant ash glasses in the early Islamic and late Bronze Age periods respectively with some success. Moreover, by comparing with v-Na-Al glass of a broad date range it helps us to understand when and where v-Na-Al glass was used and how its chemical compositions changed over time.

The present paper is part of a study on 9^th^– 16^th^ centuries AD Malindi and Mambrui glass found in the excavations of Malindi and Mambrui by a joint archaeological team from Peking University and the National Museums of Kenya in 2010–2013. It focuses on 15^th^– 16^th^ centuries AD glass vessels from Malindi and glass beads from Mambrui. A study of 9^th^– 15^th^ centuries AD glass beads from Mambrui will be published elsewhere [12].

Therefore, by analysing glass vessels and beads from 15^th^– 16^th^ century AD Malindi and Mambrui in Kenya we aim to:

- identify the raw materials that were used to produce these glasses.
- compare their chemical compositions with published data for similar glass types and therefore to refine the identification of different types of the understudied type of glass.
- suggest possible provenance(s) for v-Na-Al glass.
- identify the possible sources of the lead using lead isotope analysis and thereby the location(s) of possible glass workshop(s) that manufactured high lead and tin opaque and opaque yellow glasses.

## Materials and methods

### Archaeological sites

#### Malindi

Between 2012 and 2013, Peking University and the National Museums of Kenya formed a joint archaeological team to excavate the Malindi Old Town in central Malindi, a coastal city on the Indian Ocean in eastern Kenya [13]. During the excavations, five areas, CA, CB, CC, CD and CE were excavated. The excavations recovered more than 500 Chinese ceramic sherds, more than 1200 Islamic pottery sherds and about 70,000 local earthenware sherds. European ceramics and Indian earthenwares have also been found. Apart from ceramics, fragments of glass vessels were found.

The analysis of imported and local ceramics and radiocarbon dating confirmed 6 stages of occupational history in the Malindi Old Town [14]. The relevant stage of occupation for this study is Stage 3 (AD 1370–1520). This is the period when Malindi reached its peak, and it is at this time that Zheng He’s fleet might have arrived in the Malindi area (early 15^th^ century AD). The Portuguese led by Vasco da Gama had also landed at Malindi at the end of 15^th^ century AD.

**Fig 2:**
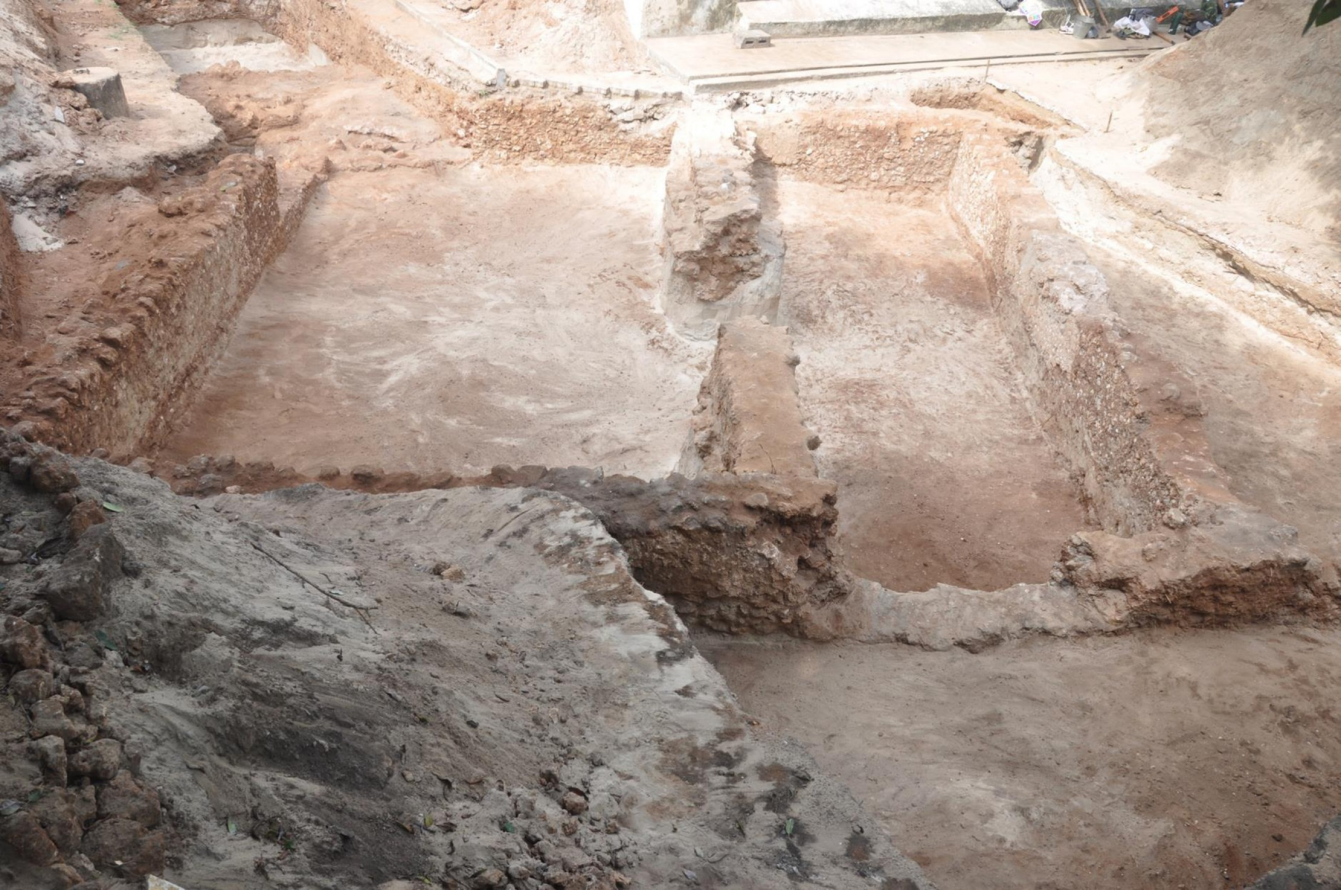
Excavated area of CA in Malindi, Kenya. Photo of the excavated Area CA in Malindi. Remains of the house foundations were found in Area CA, where all of the glass vessels were found.

The settlement was extended southwards and included Areas CA, CB and CD. Houses made of stone mixed with lime, corallite and mud were also uncovered during the excavations and are dated after the 15^th^ century AD (Fig 2) [14].

A large quantity of Chinese ceramics (e.g. Longquan celadon) and Islamic ceramics (e.g. sgraffito, black-on-yellow, monochrome blue-green glazed wares) were found in Areas B and D [13, 14]. There was a marked increase of Islamic ceramics in Malindi in this period, possibly due to an increase of trade with the Islamic east. All fragments (a total of seventeen) of glass vessels came from Area CA (where the houses were found) in trench 3 context 5. Curiously, no glass beads were found in Malindi. A number of Chinese and Islamic pottery were also found in this context. The dating of the ceramics suggests context 5 is dated to the 15^th^–early 16^th^ centuries AD [13].

#### Mambrui

A detailed description of the site and its finds has been published elsewhere [13, 14]. Here we briefly describe the site of Mambrui and its finds in the 12^th^– 16^th^ centuries AD when the majority of the v-Na-Al glass beads were imported to Mambrui.

The archaeological site of Mambrui is located in the village of Mambrui, which is located 11km from the modern city of Malindi and was excavated between 2010 and 2013 by a joint archaeological team from Peking University and the National Museums of Kenya. Twelve areas (Areas A–M) were excavated and a large quantity of remains, including house foundations, sanitary facilities, smelting and casting furnaces, walls and wells were discovered. The excavators were able to establish the functional areas of the settlement such as a central area, elite residences and craft-producing sectors [14].

**Fig 3:**
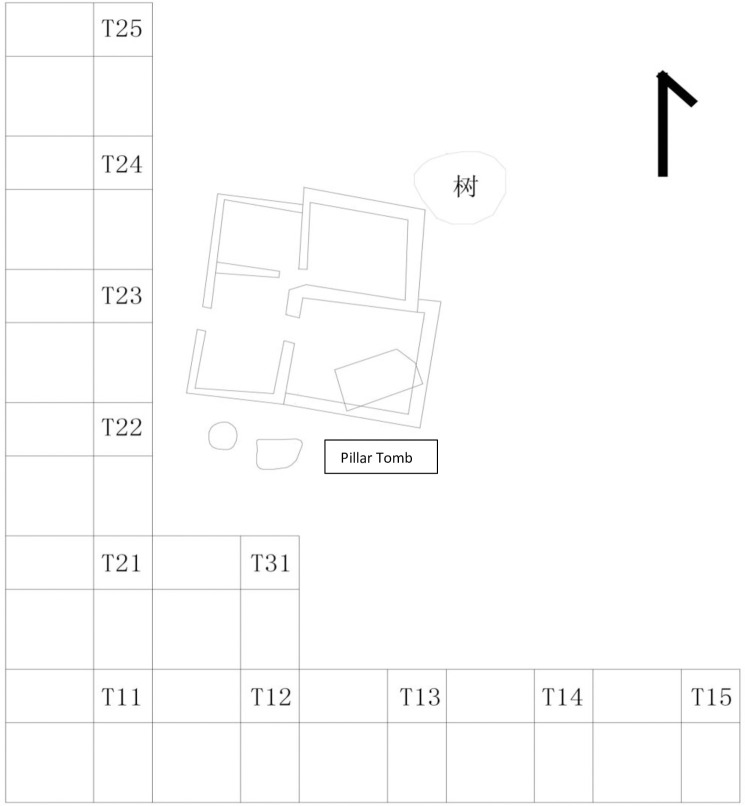
Excavated area of MA in Mambrui, Kenya. Excavation plan of Area MA in Mambrui. Most of the glass beads from Mambrui came from Area MA T12, 21 and 24.

A large quantity of artefacts was found, including more than 500 Chinese ceramic sherds, about 3000 Islamic pottery sherds, more than 130,000 local earthenware sherds and some Indian earthenware, iron slag, glass beads, and animal bones [14]. The majority of the glass beads came from Area MA trenches (T) 12, 21 and 24 contexts 2–4 (Fig 3). The associated Chinese ceramics suggests contexts 2–4 are dated to AD 1400 –AD 1520. However, glass vessels were not found at Mambrui.

During the 12^th^– 13^th^ centuries AD, the settlement of Mambrui expanded with its eastern boundary almost reaching the coastline, the Qubba Mosque was built and became the centre of the settlement. Elite stone-built residences and iron-making workshops were established near the mosque in Area E [14]. Maritime trade with countries such as China also grew substantially in this period and a substantial quantity of Chinese ceramics was imported to Mambrui.

Between AD 1275 and AD 1435 Mambrui reached its peak and the settlement continued to expand eastward and southward covering 30 ha. Trade with the Islamic east and China flourished, with large quantities of Chinese and Islamic potteries imported to the settlement. The discovery of Chinese official blue-and-white and Longquan porcelains suggests the Chinese admiral Zheng He and his fleet might have arrived in Mambrui at this time [14].

But between AD 1435 and AD 1520, Mambrui began to decline. The settlement area shrank to 15 ha and a reduced amount of Chinese ceramics in this period suggests trade between Mambrui and China had also declined. This could be the result of the Chinese ban on maritime trade after the Ming Xuande emperor’s reign (AD 1426–1435) and a decision by the Portuguese to maintain Malindi as a centre for maritime trade at the expense of Mambrui [14].

### Glass samples

**Fig 4:**
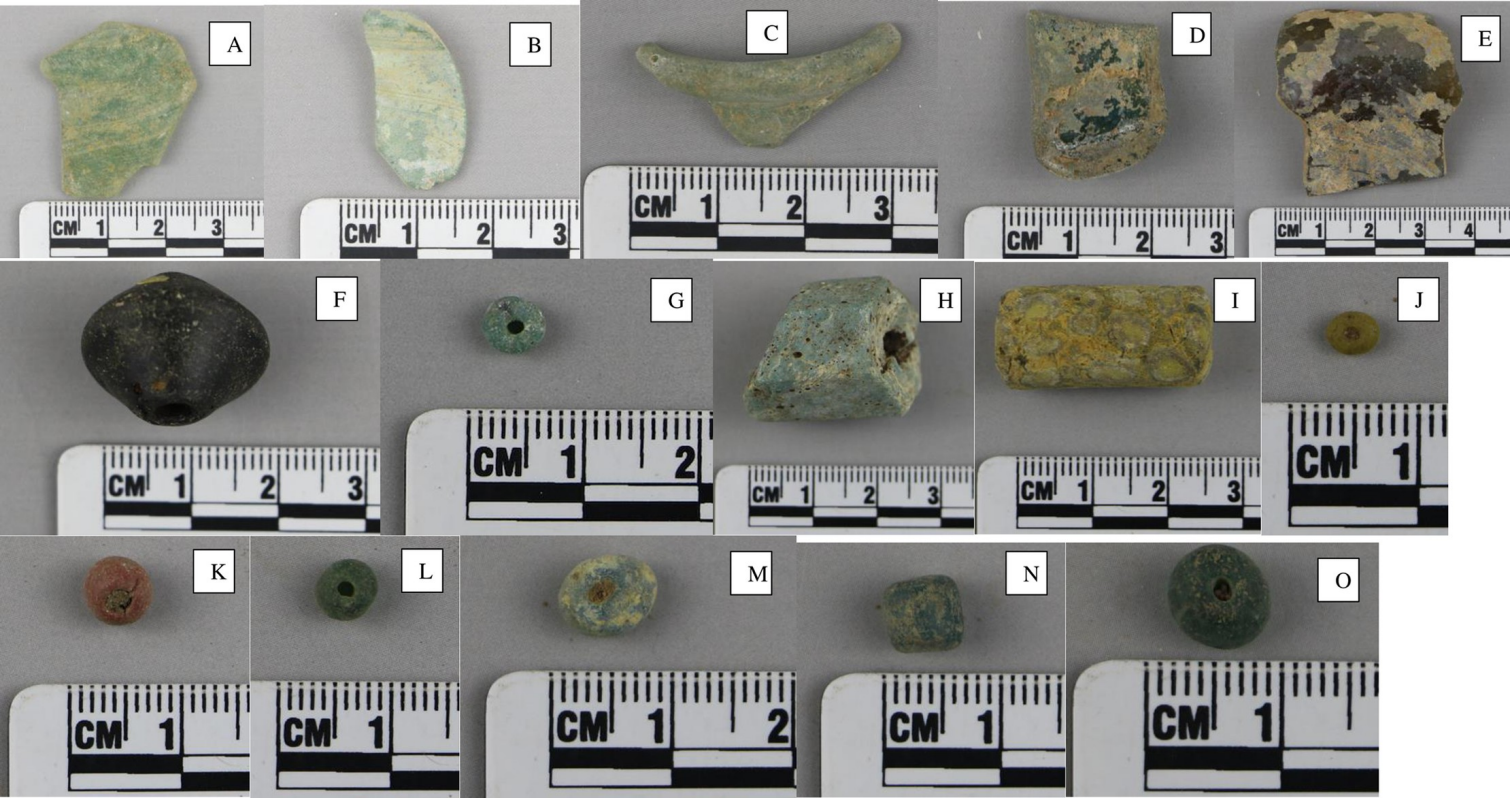
Examples of glass vessels and glass beads from Malindi and Mambrui. (**A**) A fragment of a glass bowl (G01) from Malindi; (**B**) A fragment of glass vessel (G03) from Malindi; (**C**) Rim of a bottle (G14) from Malindi; (**D**) The base of a small phial (G18); (**E**) The base of a glass bowl (G20); (**F**) A truncated bicone from Malindi (B04); (**G**) An opaque turquoise oblate bead (B28); (**H**) An octagonal rectangular prismatic bead (B41); (**I**) An opaque yellow tubualr bead (B42); (**J**) An opaque yellow oblate bead (B45); (**K**) A brownish red spheroid bead (B50); (**L**) A green tubular bead (B52); (**M**) A light green oblate bead (B59); (**N**) A green tubular bead (B60); (**O**) A green oblate bead (B64).

All of the glass vessel fragments (a total of 17) from Malindi were selected for analysis. The glass beads were selected on the basis of shapes and colours and the authors tried to include all possible glass bead types present in the assemblage. All of the glass samples from the Malindi excavations were selected for electron microprobe analysis (EPMA-WDS) and trace element analysis using laser ablation-inductively coupled plasma-mass spectrometry (LA-ICP-MS). Based on the dating of the Chinese and Islamic ceramics found in the same context, Area CA context T3 (5), and radiocarbon dating, the glass vessels are dated to the 15^th^–early 16^th^ centuries AD [13] (Fig 4A–4E). They were coloured in various shades of green (Table 1). Twenty glass beads (Fig 4F–4O) from the Mambrui excavations were selected only for trace element analysis using LA-ICP-MS. They are dated to the 15^th^– 16^th^ centuries AD and various colours, including various shades of green, turquoise and yellow were selected for this study (Table 2). The glass samples are currently housed in the School of Archaeology and Museology in Peking University, China and permission is required for future access to the materials. All necessary permits were obtained for the described study, which complied with all relevant regulations.

**Table 1:** Description of the glass vessels from Malindi, Kenya.

Specimen Numbers	Area-Context	Date	Vessel Forms	Colour
G01	CA-T3 (5)	15^th^–early 16^th^ centuries AD	Bowl fragment	Light green translucent
G02	CA-T3 (5)	15^th^–early 16^th^ centuries AD	Vessel fragment	Light green translucent
G03	CA-T3 (5)	15^th^–early 16^th^ centuries AD	Vessel fragment	Light green translucent
G05	CA-T3 (5)	15^th^–early 16^th^ centuries AD	Vessel fragment	Light green translucent
G06	CA-T3 (5)	15^th^–early 16^th^ centuries AD	Vessel fragment	Light green transparent
G07	CA-T3 (5)	15^th^–early 16^th^ centuries AD	Vessel fragment	Green transparent
G08	CA-T3 (5)	15^th^–early 16^th^ centuries AD	Vessel fragment	Light green translucent
G09	CA-T3 (5)	15^th^–early 16^th^ centuries AD	Vessel fragment	Light green translucent
G10	CA-T3 (5)	15^th^–early 16^th^ centuries AD	Vessel fragment	Light green translucent
G11	CA-T3 (5)	15^th^–early 16^th^ centuries AD	Vessel fragment	Light green translucent
G12	CA-T3 (5)	15^th^–early 16^th^ centuries AD	Vessel fragment	Light green translucent
G13	CA-T3 (5)	15^th^–early 16^th^ centuries AD	Bowl fragment	Green translucent
G14	CA-T3 (5)	15^th^–early 16^th^ centuries AD	Small bottle rim	Green translucent
G15	CA-T3 (5)	15^th^–early 16^th^ centuries AD	Vessel fragment	Dark green (‘black’)
G16	CA-T3 (5)	15^th^–early 16^th^ centuries AD	Small bottle rim	Light green translucent
G18	CA-T3 (5)	15^th^–early 16^th^ centuries AD	Small phial base	Green translucent
G20	CA-T3 (5)	15^th^–early 16^th^ centuries AD	Bowl base	Yellow-green translucent

They are in the forms of bottles and bowls and are coloured in various shades of green.

**Table 2:** Description of the glass beads from Mambrui, Kenya.

Specimen Numbers	Area-Context	Date	Shape	Colour	How it was made	Length x diameter
B04	Heka-1c-T4	AD 1400–1520	Truncated bicone	Dark green opaque	Wound	16mm x 6mm
B28	MD-DT1	AD 1400–1520	Oblate	Green	Drawn	2.5mm x 4.5mm
B41	MA-T25 (2)	AD 1400–1520	Octagonal rectangular prismatic	Turquoise opaque	Drawn	25mm x 20mm
B42	MA-T21 (3)	AD 1400–1520	Tube	Yellow opaque	Drawn	28mm x 14mm
B45	MA-T21:F4	AD 1400–1520	Oblate	Yellow opaque	Drawn	1mm x 3mm
B50	T12 3	AD 1400–1520	Spheroid	Brownish red opaque	Wound	4mm x 4mm
B52	MA-T24 (2)	AD 1400–1520	Tube	Green Translucent	Drawn	4mm x 4mm
B54	MA-T24 (2)	AD 1400–1520	Tube	Brownish red opaque	Drawn	4mm x 2mm
B57	MA-T24 (2)	AD 1400–1520	Tube	Green	Drawn	5mm x 4mm
B58	MA-T24 (2)	AD 1400–1520	Oblate	Green	Drawn	5mm x 4mm
B59	MA-T24 (2)	AD 1400–1520	Oblate	Light green	Drawn	5mm x 6mm
B60	MA-T24 (2)	AD 1400–1520	Tube	Green	Drawn	6mm x 5mm
B61	MA-T24 (2)	AD 1400–1520	Tube	Light green	Drawn	6mm x 8mm
B63	MA-T24 (2)	AD 1400–1520	Oblate	Green	Drawn	4mm x 5mm
B64	MA-T24 (2)	AD 1400–1520	Oblate	Green	Drawn	6mm x 5mm
B65	MA-T24 (2)	AD 1400–1520	Oblate	Green	Drawn	4mm x 5mm
B66	MA-T24 (2)	AD 1400–1520	Oblate	Green	Drawn	3mm x 7mm
B67	MA-T24 (2)	AD 1400–1520	Oblate	Green	Drawn	3mm x 4mm
B68	MA-T24 (2)	AD 1400–1520	Spheroid	Green	Wound	5mm x 4mm
B72	MA-T24 (2)	AD 1400–1520	Oblate	Green	Drawn	3mm x 4mm

All of the glass beads are wound and drawn beads and come in different shapes include oblate, spheroid, tube and bicone.

### Analytical methods

#### Electron probe microanalysis

The glass samples were mounted in cold-setting epoxy resin, and then were ground and polished using standard sample preparation procedures down to a 0.02 μm final polishing solution. The samples were coated with a thin film of carbon of 25 μm prior to analysis, to allow the conduction of the electron beam. The polished samples were analysed by EPMA–WDS, using a JEOL JXA- 8200 electron microprobe in the University of Nottingham Nanoscale and Microscale Research Centre (nmRC). Quantitative compositional analyses were carried out using the following analytical set-up: 20kV accelerating voltage, 50nA beam current and a 50 μm defocused beam. The counting times were 30s on the peak and 15s on the background to either side of the peak. A defocused beam is used to reduce the effect of the migration of alkalis within the samples [15]. The EPMA–WDS was calibrated against a combination of certified standard reference materials, including minerals (orthoclase, jadeite, pyrite, wollastonite and MgO), pure metals (Mn, Ti, Cu, Ag, V, Sb, Zn, Sn, Ni, Co., Cr and Zr) and synthetic standards (PbTe, GaP, InAs, KCl, BaF and SrF), and was corrected using phi-rho-z model. The compositions of 22 elements were sought and were expressed as weight percentage oxides. Three areas of interest were analysed in each sample and the mean and standard deviation were calculated. So as to check the accuracy and precision of the EPMA–WDS system and to monitor any drift in the instrument [16], six analyses of a secondary standard, Corning B, were included during the analytical run (two sets of analyses at the start and another two sets at the end of the sample run, and another two sets of analyses half way through the analytical run; for the results, see S1 Table).

#### Laser ablation-inductively coupled plasma mass spectrometry

The glass samples from Malindi and Mambrui were mounted in cold-setting epoxy resin, and then were ground and polished using standard sample preparation procedures down to a 0.02 μm final polishing solution. The polished samples were analysed by laser-ablation-inductively coupled plasma mass spectrometry (LA-ICP-MS) in Beijing Createch Testing in Beijing, China to determine the major, minor and trace elements in the samples. A NewWave UP193 nm excimer system and an Analytikjena PlasmaQuant MS Elite model ICP-MS type were used. The laser was operated at an energy density of 2.04 J/cm^2^, a pulse frequency of 10 Hz and with a beam diameter of 35 μm. Helium is advantageous as a carrier gas and was applied in this study [17]. Prior to analysis glass standard NIST SRM 610 was used to calibrate the instrument to achieve an optimal state. A 20s pre-ablation time was followed by 45s analytical time. We have adopted the procedure described by Liu *et al*. [17] in which by normalising the sum of all metal oxides to 100% and calibrating them against multiple external standards, we can precisely determine major and trace elements without the need for applying internal standards. Five external standards, NIST 610, NIST 612, BHVO-2G, BCR-2G and BIR-1G, were used for external calibration and to calculate the quantitative concentrations for forty-nine elements. They were measured every ten sample sets (for the results, see S2 Table). Certified values provided by NIST was limited to only a number of values, therefore concentrations from Pearce *et al*. [18] were used for other elements. The software ICPMSData Cal. was used to process the data (including correction of instrument sensitivity drift, calculation of element contents). We have used the Si values from the EPMA to calibrate the LA-ICP-MS data for the Malindi glass vessels and the oxides of the glass beads are normalised to 100% assuming that the sum of their concentrations in weight percent in glass is equal to 100% [19].

#### Lead isotope analysis

Lead isotope ratios were measured using a multi-collector inductively coupled plasma mass spectrometer (MC-ICP-MS) of the type VG Elemental in the School of Earth and Space at Peking University. Small fragments of samples were taken from the glass beads and were dissolved in nitric acid, leaching lead into the solutions. The clear solution is then diluted with deionised water. We used ICP-AES to measure the lead contents in the solutions which are then diluted down to the tolerance limit (1 ug/I) of the instrument. Finally, the thalium (Tl) standard SRM997 were then added into the solutions [20, 21]. Repeated analyses of SRM981 were conducted during the analytical run to check the accuracy and precision of the instrument (at the start and end of each sample set) (for the result, see S3 Table).

## Results and discussion

### Results

#### Chemical analysis

The full EPMA and LA-ICP-MS results are given in S5 Table and the means and standard deviations for selected oxides and elements in the glass samples are presented in Table 3. S4 Table presents reduced compositions for all samples which are indicated with a * in all graphs and tables where applicable. For the glass vessels, we have used the EPMA results for the major and some minor element oxides (Na_2_O, SiO_2_, CaO, MgO, K_2_O and Al_2_O_3_) and the LA-ICP-MS results for other minor and for trace elements. For the glass beads, we have used the LA-ICP-MS results for the major, minor and trace elements. The glasses are all of a soda-lime-silica glass type. The high concentrations of K_2_O* (2.34% - 4.75%) and MgO* (3.17% - 5.13%) in the glasses suggest plant ash was the primary alkali flux. All glasses have high concentrations of Al_2_O_3_* (3.28% - 9.96%), which suggests that impure sand was the main silica source [8, 22 (p. 114)]. The CaO* and SiO_2_* contents vary between 3.94% and 7.90% and between 54.24% and 68% respectively.

**Table 3:** The means and standard deviations of Malindi glass vessels and Mambrui glass beads.

Elements	Malindi glass vessels (n = 17)	Green Mambrui glass beads (excluding B04) (n = 14)	Opaque red Mambrui glass beads (n = 2)
SiO_2_	61.89% ± 1.49%	61.73% ± 1.33%	60.90 ± 0.56
Na_2_O	15.86% ± 0.93%	16.30% ± 1.14%	15.25 ± 0.63
CaO	5.48% ± 1.02%	5.59% ± 0.95%	5.47 ± 0.38
MgO	4.41% ± 0.30%	4.51% ± 0.54%	3.82 ± 0.02
K_2_O	3.10% ± 0.28%	3.11% ± 0.56%	3.74 ± 0.38
Al_2_O_3_	5.67% ± 0.39%	5.61% ± 0.29%	5.55 ± 0.26
FeO	1.15% ± 0.22%	1.32% ± 0.49%	2.47 ± 0.62
MnO	0.08% ± 0.03%	0.07% ± 0.02%	0.09 ± 0.05
P_2_O_5_	0.42% ± 0.12%	0.40% ± 0.07%	0.42 ± 0
Ti (ppm)	2081 ± 430	2125 ± 432	1828 ± 370
Li (ppm)	8 ± 2	11 ± 4	12 ± 0.6
B (ppm)	146 ± 14	163 ± 21	147 ± 8
Cr (ppm)	23 ± 5	22 ± 3	19 ± 7
Co (ppm)	14 ± 22	82 ± 177	13 ± 9
Cu (ppm)	599 ± 629	6906 ± 2330	11133 ± 363
Zn (ppm)	90 ± 126	50 ± 26	135 ± 124
Sr (ppm)	377 ± 56	365 ± 49	369 ± 23
Zr (ppm)	69 ± 10	70 ± 13	67 ± 9
Sn (ppm)	293 ± 547	178 ± 157	657 ± 382
Sb (ppm)	6 ± 8	13 ± 5	17 ± 13
Cs (ppm)	0.3 ± 0.1	0.2 ± 0.1	0.2 ± 0
Ba (ppm)	417 ± 21	406 ± 15	412 ± 15
La (ppm)	6 ± 1	6 ± 1	6 ± 1
Pb (ppm)	1363 ± 2111	554 ± 729	4011 ± 2995
U (ppm)	0.4 ± 0.1	0.4 ± 0.1	0.4 ± 0.1

The results are displayed according to glass forms and colours. Single samples are not listed. Only selected oxides (in wt %) and elements (in ppm) are presented here.

The high concentrations of alumina, potash and magnesia, and the relatively low level of calcium in some glasses show that they belong to the high alumina-plant ash (v-Na-Al) glass [4, 8, 9, 23, 24].

Two glass beads, B04 and B42, are notably different from other glass vessels and beads. B04 has a higher concentration of Al_2_O_3_* (9.96%) and trace elements associated with the silica source such as Zr (112.67 ppm), Ti (4912.78 ppm), Nd (21.09 ppm), La (25.11 ppm), V (50.75 ppm) and Cr (46.24 ppm). On the other hand, B42 has a rather low concentration of Al_2_O_3_* (3.28%), Ba (165.22 ppm) and Sr (255.72 ppm) as well as an elevated level of La (15 ppm), Nd (12.09 ppm) and V (30.14 ppm) that are associated with the silica source. This suggests B04 and B42 were manufactured with sand sources different from other glass samples in the assemblage. Therefore, glass beads B04 and B42 will be excluded from further discussion in this paper which focuses solely on v-Na-Al glass.

Generally speaking, the chemical compositions of most of the vessel and bead glasses from both sites is quite homogenous which could suggest a single source (S4 Table). Apart from glass beads B04 and B42, it is noted that a number of glass beads and glass vessels have elevated levels of Na_2_O* (>16%) and CaO* (>6%) (S4 Table). The main compositional difference between the vessels and beads is the elements associated with coloration and opacification, Sn, Sb, Co, Cu and Pb, which are higher in the beads (Table 3).

The limited compositional variation includes trace elements associated with the silica used. There is a fairly low compositional difference between the glass vessels and beads related to the sand source used. They all have a relatively low concentration of Zr (avg. 69 ppm for both groups) and Ti (avg. 2081 ppm and 2084 ppm) and the two elements in the beads and vessels are positively correlated. Levels of Cr and La are also relatively low (avg. 23 ppm and 21 ppm; avg. 6 ppm for both groups respectively), but the concentrations of Ba are relatively high, with averages of 417 ppm and 407 ppm in the vessels and beads respectively.

The glass vessels and the majority of the glass beads are coloured in variety of green hues, ranging from light to dark green (Tables 1 and 2). All of the glass vessels have high concentrations of FeO* (0.76% - 1.61%), which is most likely derived from the impurities of sands. The green colour is produced by the presence of iron in the glass often as mixed ferric (Fe^3+^) and ferrous (Fe^2+^) ions [25, 26].

The opaque red glass bead (B50) contains high levels of Cu (11390 ppm) and FeO* (2.09%). The iron in the glass helps to reduce the copper cation, and by heat treating the glass the copper particles can be ‘struck’ from the glass matrix, imparting a red colour [27]. The high concentrations of lead (6128 ppm) and tin (927 ppm) shows that a tin-based opacifier is present. Glass bead B41 contains a high concentration of Cu (14145 ppm): cupric oxide (CuO) is present in the glass and produces a turquoise blue colour [26]. Elevated to high concentrations of lead and elevated to high concentrations of tin in B41, B45 and B50 suggest that a tin-based opacifier (lead stannate) is present [5]. The level of lead in these beads vary between 6128 ppm and 75783 ppm and the levels of tin between 927 ppm and 28864 ppm. Further examination with an SEM with BSE imaging and XRD would help to investigate this further.

#### Lead isotope analysis

Four glass beads, B41, B45 and B50 from Mambrui were selected for lead isotope analysis because of their high lead contents (for the full results, see S6 Table). The lead contents were most likely introduced into the glass beads as colourants and opacifiers. Therefore, the lead isotope result reflects the production area of the colourants and opacifiers [28]. The lead isotope results show that the glass beads have relatively homogenous lead isotope ratios: ^208^Pb/^206^Pb ratios ranging from 2.0694–2.0945, ^207^Pb/^206^Pb ratios between 0.8362 and 0.8445 and ^206^Pb/^204^Pb ratios between 18.531–18.686.

### Discussion

The glass vessels from Malindi and the glass beads from Mambrui can be confirmed as plant ash high alumina (v-Na-Al) glass, which has been found in Central Asia [29], southeast Asia (e.g. the Island of Sumatra in Indonesia and Malaysia), Mtwapa in Kenya and southern Africa, with rare examples occurring in the west [30]. It is characterised by high Al_2_O_3_ and relatively low SiO_2_ and CaO [4, 8, 9, 23, 24]. It is dated to the 9^th^– 16^th^ centuries AD and it became more common in southeast Asia during the 12^th^– 13^th^ centuries AD and in Africa during the 13^th^– 16^th^ centuries AD [8, 9, 23, 24].

Despite a lack of analytical research on sub-Saharan African glass vessels, considerable progress has been made on African glass beads, with v-Na-Al glass beads having been found in southern Africa (Mapungubwe and Zimbabwe beads series, and Madagascar). A comparison between our data and published compositions can shed light on the raw materials, production zones and provenance of Malindi and Mambrui glass. By comparing with v-Na-Al glasses of a broad date range, the authors believe that it will help us to understand when and where v-Na-Al glass were used and how their chemical compositions changed overtime. Without looking at v-Na-Al glass from an earlier time period, it is difficult to redefine v-Na-Al glass groups in a way that puts the Malindi and Mambrui glass into a wider developmental context.

**Fig 5:**
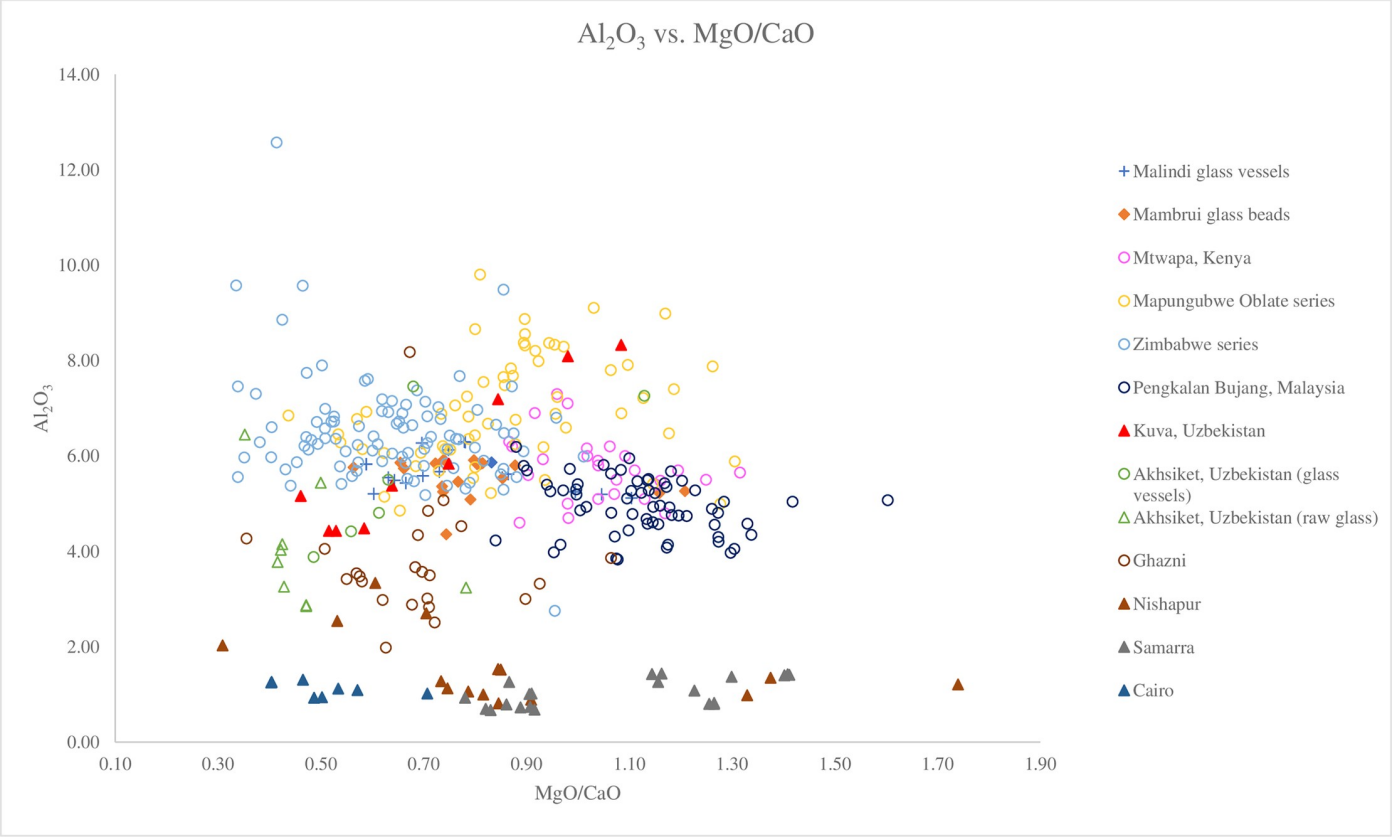
A biplot of Al_2_O_3_ versus MgO/CaO for Malindi glass vessels and Mambrui glass beads with relevant 9^th^– 16^th^ centuries AD v-Na-Al glass found in Africa and southeast Asia and low alumina soda glass from the Middle East. The data is displayed according to compositional groups and sites. Data are from Mtwapa [8]; Mapungubwe Oblate series and Zimbabwe series from southern Africa [4]; Pengalan Bujang [9]; Ghazni [36]; Kuva and Akhsiket [29]; Nishapur, Samarra and Cairo [10]. It shows that Malindi, Mambrui, v-Na-Al and Central Asian glasses are distinguishable from Middle Eastern plant ash glass from Nishapur, Samarra and Cairo with higher levels of Al_2_O_3_.

While lower alumina soda plant ash glass from the Middle East has been found in Africa, which suggests a glass-trading network between Africa and the Middle East existed as early as the 8^th^ century AD [2, 4], the high concentrations of Al_2_O_3_ in the Malindi and Mambrui glass means (averages of 5.67% and 5.55% respectively) rules out the possibility that they were made in western Asia in centres such as Damascus in Syria, Tyre in Lebanon, Banias in Palestine, al-Raqqa in Syria, Samarra in Iraq, Nishapur in Iran. Plant ash glass from these centres contains relatively low concentrations of Al_2_O_3_ of between 0.5% and 3.5%, with some containing up to c. 4% [10, 31–35] (Fig 5). The trace elements of such Middle Eastern glasses are also distinct from v-Na-Al glass (see below).

#### Plant ash

It has been demonstrated by Barkoudah and Henderson [37] and Henderson [22] that the levels of MgO and CaO in plants are primarily determined by the geology of the environment where the plants were grown; while the alkali levels (particularly K_2_O and Na_2_O) in the plants are determined by the physiology of plant species and plant genera [22, 37]. Trace elements such as Rb, Li and Cs tend to be associated with the source of alkalies used in glasses [10].

**Fig 6:**
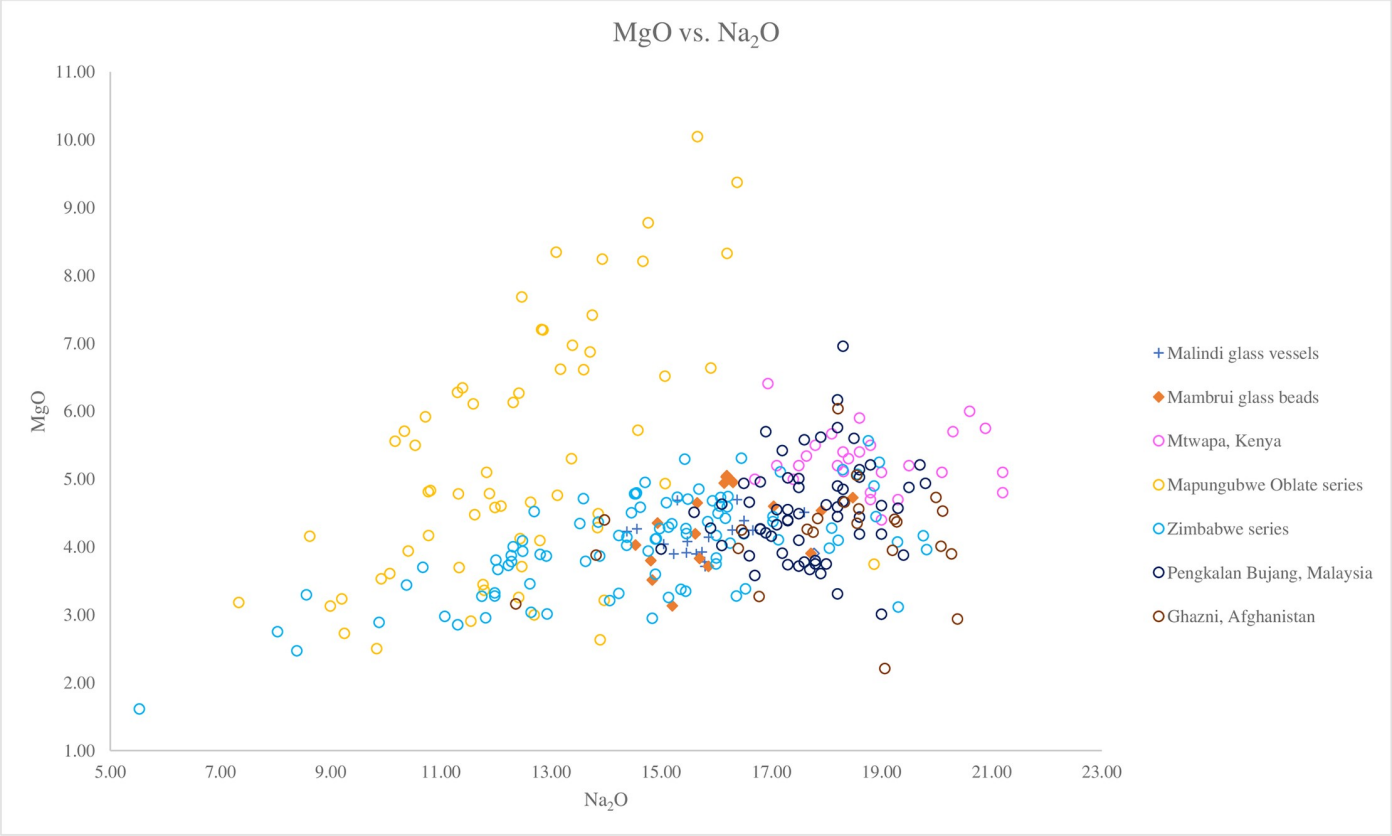
A biplot of MgO versus Na_2_O for Malindi glass vessels and Mambrui glass beads with relevant 9^th^– 16^th^ centuries AD v-Na-Al glass found in Africa and southeast Asia: Mtwapa [8]; Mapungubwe Oblate series and Zimbabwe series from southern Africa [4]; Pengalan Bujang [9] and Ghazni [36]. The figure shows that the Malindi glass vessels and Mambrui glass beads have higher Na_2_O than the Mapungubwe Oblate glass beads, which might reflect the use of different sources of plant ashes.

**Fig 7:**
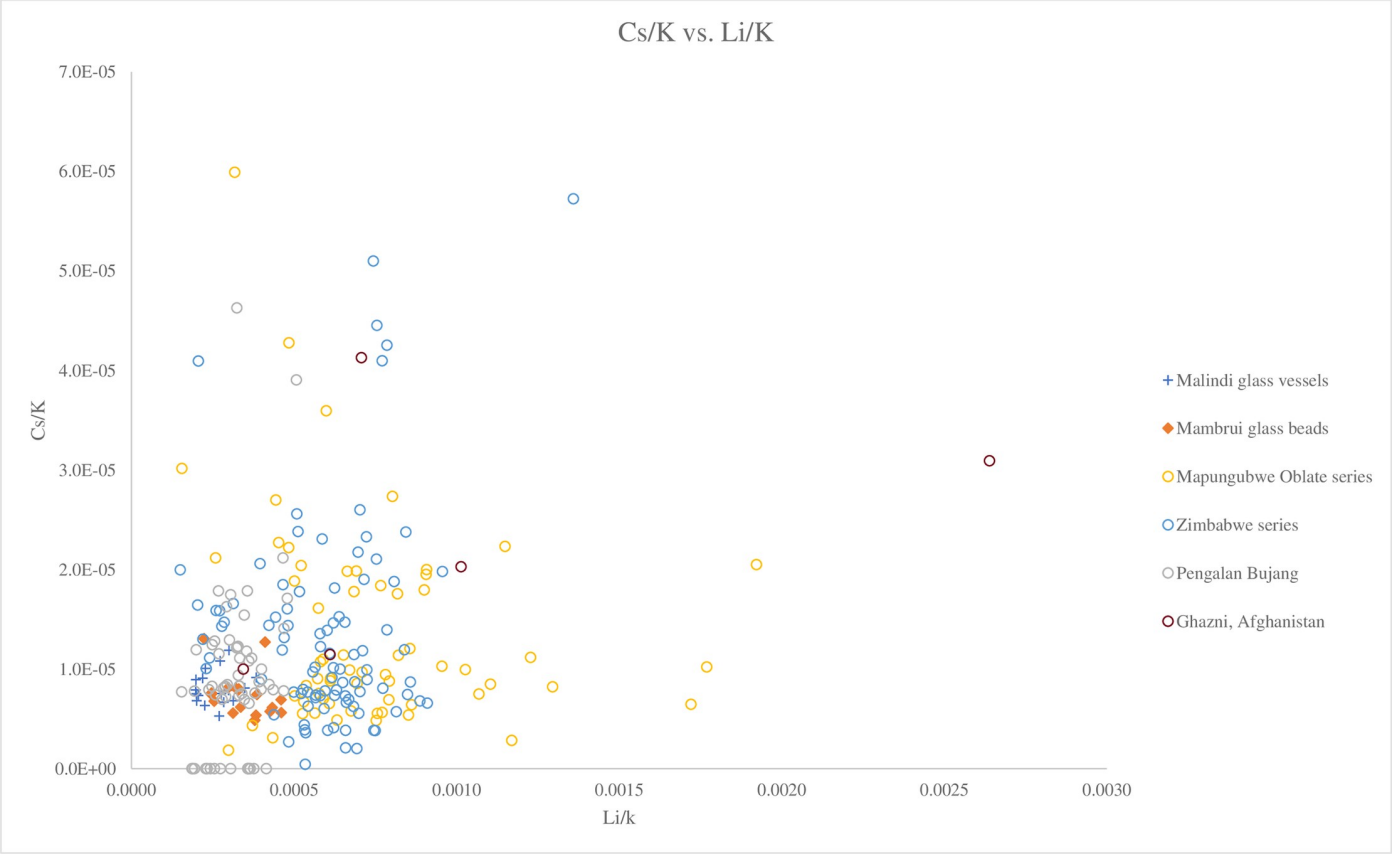
A biplot of Cs/K versus Li/K for Malindi glass vessels and Mambrui glass beads with relevant 9^th^– 16^th^ centuries AD v-Na-Al glass found in Africa and southeast Asia. The data is displayed according to compositional groups and sites. Data are from Mtwapa [8]; Mapungubwe Oblate series and Zimbabwe series from southern Africa [4]; Pengalan Bujang [9] and Ghazni [36]. It can be seen that Malindi and Mambrui glasses are distinguishable from Mapungubwe Oblate glass beads with lower Li/K ratios. This suggests different plant species and/or genera from different geological environments were used to produce Malindi and Mambrui glasses.

Fig 5, a plot of Al_2_O_3_ versus MgO/CaO, shows that the Malindi glass vessels and Mambrui glass beads have similar ratios of MgO/CaO and are similar to the Zimbabwe bead series, a group of Mapungubwe Oblate series with low MgO/CaO ratio and the beads from Madagascar. What distinguishes between these African glasses compositionally is their Na_2_O levels (Fig 6). The Malindi and Mambrui glass and the Zimbabwe series have a higher concentration of Na_2_O (avg. 15.86%, 16.08% and 14.56% respectively) compared to the Mapungubwe Oblate series and the Madagascar glass beads (avg. 13.47% and 13.01% respectively), which may suggest different plant species and/or genera from different geological environments were used to produce Malindi and Mambrui glasses. Trace element analysis also suggests that different sources of plants were used. Fig 7 shows that Malindi and Mambrui glasses are distinguishable from southern African and Madagascar glass beads, some with lower Li/K ratios.

The Malindi and Mambrui glass appears to be different from the glass vessels from Mtwapa in Kenya, a settlement on the southern Kenyan coast where v-Na-Al glass vessels were found [8]. Much of the Mtwapa glass has higher concentrations of soda and/or magnesia than the Malindi and Mambrui glass (Fig 6). It also has a generally higher MgO/CaO ratios than the Malindi and Mambrui glass (Fig 5), which means a different type of plant ash, characterised by a relatively high soda and magnesia contents was used to produce the Mtwapa glass vessels. The same applies to the southeast Asian glass from Pengalan Bujang in Malaysia, most of which contains lower MgO/CaO ratios and higher levels of Na_2_O (avg. 17.68%) than the Malindi and Mambrui glass (Figs 5 and 6). Surprisingly, a group of glass from Pengalan Bujang has the same or similar Li/K and Cs/K ratios to the Malindi and Mambrui glass (Fig 7).

#### Sand source

The high concentrations of Al_2_O_3_ (>5%) in v-Na-Al glass, along with high concentrations of impurities such as TiO_2_ and FeO, shows that an impure sand was used to produce this type of glass. However, using major and minor elements (e.g. SiO_2_, Al_2_O_3_ and FeO) to distinguish between different sand sources proves difficult. Trace elements such as Zr, Ti, Ba, Cr and La, which derive mainly from the siliceous matrices (quartz/sand and clay) of glass, are increasingly used to distinguish between different sand sources and provide further insights into the raw materials used in glassmaking [10, 11, 37].

Zr, Ti, Ba, Cr and La can be found in various minerals in rocks or sediments such as zircon (Zr), rutile (Ti), ilmenite (Ti), monazite (La), chromite (Cr) and barite (Ba). The variation of their concentrations reflects the local geology of the sand precursors and allows us to differentiate sand sources used to make glass [38, 39].

**Fig 8:**
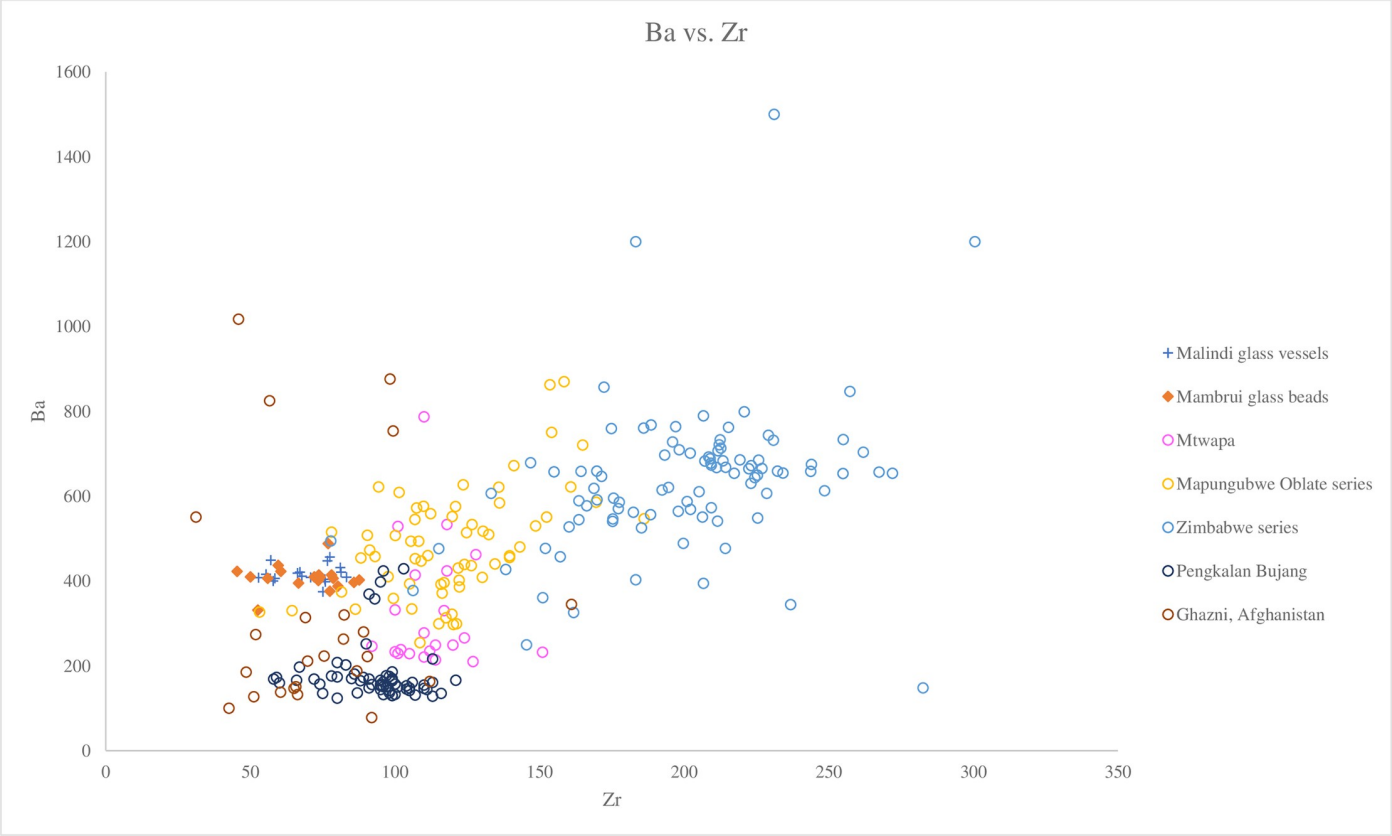
A biplot of Ba versus Zr for Malindi glass vessels and Mambrui glass beads with relevant 9^th^– 16^th^ centuries AD v-Na-Al glass found in Africa and southeast Asia. The data is plotted according to compositional groups and sites. Data are from Mtwapa [8]; Mapungubwe Oblate series and Zimbabwe series from southern Africa [4]; Pengalan Bujang [9] and Ghazni [36]. It shows that the Malindi and Mambrui glasses have similar level of Ba to the Mapungubwe Oblate glass beads but that the difference in Zr concentrations reflects local variations in the mineralogy of the sands used. The difference in the levels of Ba between the Malindi and Mambrui glass beads and glasses from Mtwapa in Kenya and Pengalan Bujan in Malaysia suggests the use of different sand sources.

**Fig 9:**
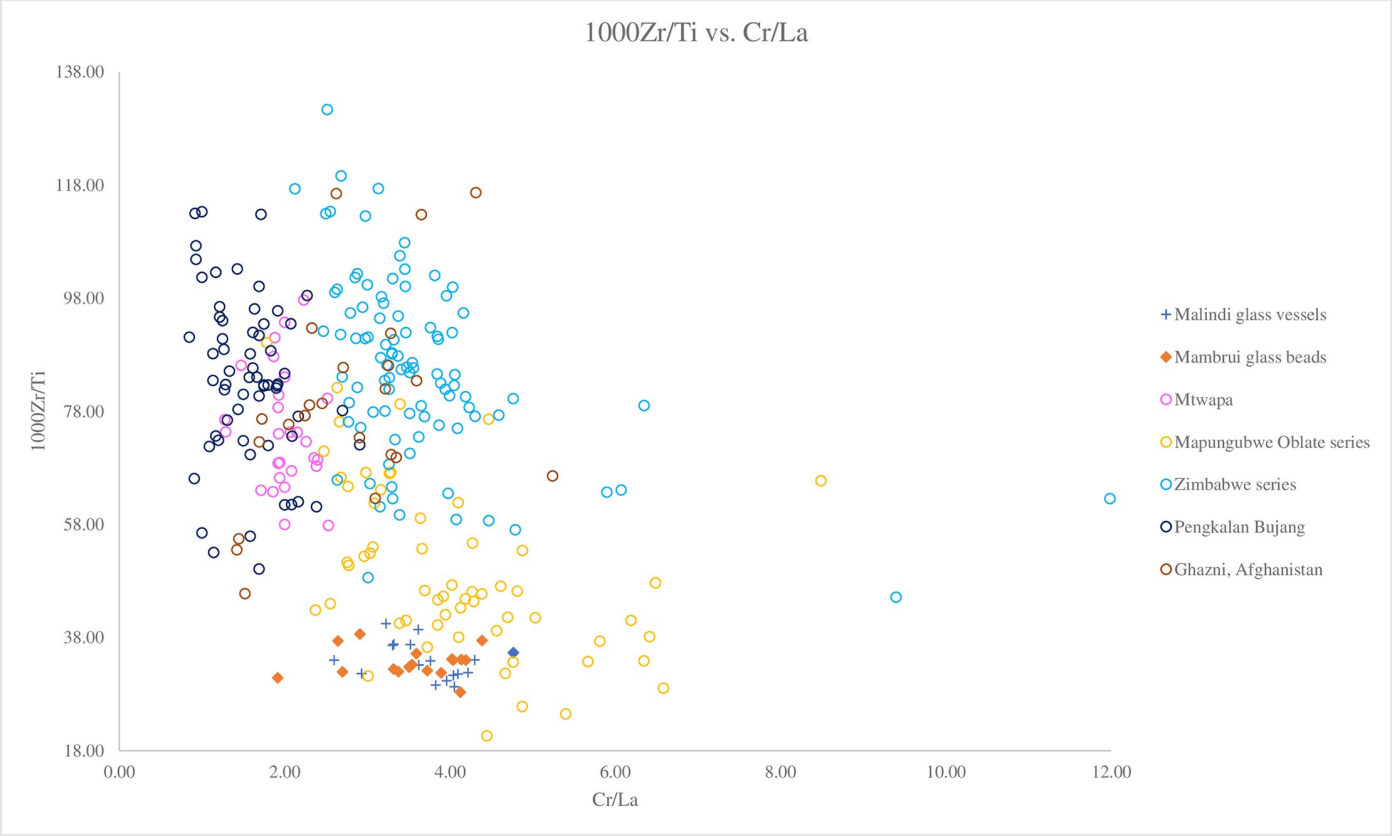
A biplot of 1000Zr/Ti versus Cr/La for Malindi glass vessels and Mambrui glass beads with relevant 9^th^– 16^th^ centuries AD v-Na-Al glass found in Africa and southeast Asia. The data is displayed according to compositional groups and sites. Data are from Mtwapa [8]; Mapungubwe Oblate series and Zimbabwe series from southern Africa [4]; Pengalan Bujang [9] and Ghazni [36]. It can be seen that Malindi and Mambrui glasses plot close to the Mapungubwe Oblate glass beads with low 1000Zr/Ti and Cr/La. They can be distinguished from the Zimbabwe series glass beads that have high 1000Zr/Ti ratios but similar Cr/La ratios.

**Fig 10:**
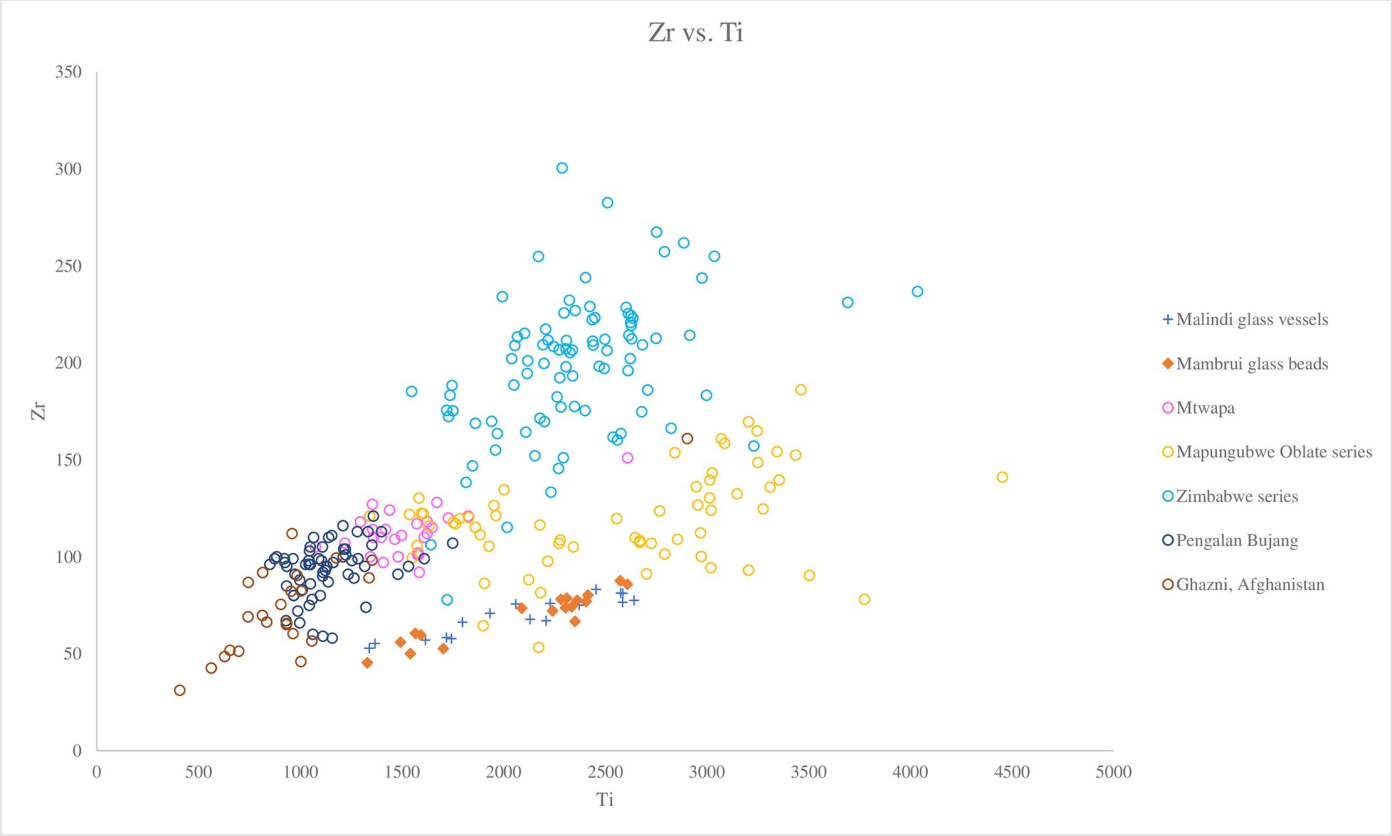
A biplot of Zr versus Ti for Malindi glass vessels and Mambrui glass beads with relevant 9^th^– 16^th^ centuries AD v-Na-Al glass found in Africa and southeast Asia. The data is displayed according to compositional groups and sites. Data are from Mtwapa [8]; Mapungubwe Oblate series and Zimbabwe series from southern Africa [4]; Pengalan Bujang [9] and Ghazni [36]. The figure shows that the Malindi and Mambrui glasses plots close to the Mapungubwe Oblate series with low Zr and Ti concentrations. The low level of Zr in Malindi and Mambrui glass compared to the Mapungubwe Oblate series suggests that the former was produced using a similar sand source to the latter but that the difference in Zr concentrations reflects local variations in the mineralogy of the sands used. They also share a similar characteristic to Ghazni glass from Afghanistan.

We can distinguish between different groups of v-Na-Al glasses using Ba versus Zr. Fig 8 shows that the Malindi and Mambrui glasses plot closely to the Mapungubwe Oblate series, which also has low concentrations of Ba (avg. 486 ppm) and Zr (avg. 119 ppm). They are markedly different from the Zimbabwe series, which is characterised by higher concentrations of Zr (avg. 200 ppm) and mainly higher Ba (avg. 635 ppm) (Fig 8). Trace elemental ratios (Cr/La versus 1000Zr/Ti) also show that the Malindi and Mambrui glasses plot close to the Mapungubwe Oblate series, with lower ratios of 1000Zr/Ti than the Zimbabwe series (45.15–131.33) but similar Cr/La ratios (Fig 9). However, looking at Fig 10, a biplot of Zr versus Ti, it can be seen that the Malindi and Mambrui glass has a rather low level of Zr (avg. 69 ppm for both groups) compared to the Mapungubwe Oblate series (avg. 119.10 ppm).

We suggest that the Malindi and Mmabrui glasses were produced using a similar sand source to the Mapungubwe Oblate series glass beads but that the differences in Zr concentrations reflects local variations in the mineralogy of the sands used. The Zimbabwe series appears to have been produced with an entirely different source of sands characterised by higher concentrations of Zr and Ba and lower ratios of 1000Zr/Ti.

Further comparison with Mtwapa glass vessels also shows a marked difference from the two Kenyan glass assemblages. The majority of Mtwapa glass has a lower concentration of Ba and an elevated level of Zr (avg. 112 ppm) [8]. Fig 9 also shows that Mtwapa glass has lower Cr/La ratios (1.20–2.52) and higher 1000Ti/La ratios (57.83–98.47) than Malindi and Mambrui glasses. This therefore shows that it is unlikely that Malindi and Mambrui glasses were produced using the same raw materials as Mtwapa glass.

The Malindi and Mambrui glasses are distinct from southeast Asian glass vessels from Pengalan Bujang in Malaysia. Most of the Malaysian glass is characterised by a relatively low level of Ti (1136 ppm), much lower Ba (179 ppm) and higher 1000Zr/Ti ratios compared to the Malindi glass (Figs 8–10). This suggests they were produced from sand sources with different mineralogical contents in different locations.

Therefore, there is a high probability that the Malindi and Mambrui glasses share a similar glassmaking tradition to the Mapungubwe Oblate series. They are characterised by an elevated level of Ti and Ba and most have overlapping of Cr/La ratios, and lower ratios of 1000Zr/Ti. However, the Mapungubwe oblate bead compositions display a considerably wider compositional variation. The differences in the concentration of Zr, Li/K, CaO and Na_2_O suggests that the Kenyan glasses are a subgroup of the Mapungubwe Oblate beads compositions and that they may have come from a different glass workshop which specialised in the production of this particular v-Na-Al compositional group.

#### Compositional groups of v-Na-Al glass

Based on the analysis above and previous research on v-Na-Al glass, the authors have tentatively identified four possible compositional groups of v-Na-Al glass dating to between the 9^th^ and 16^th^ centuries AD. These have been compared with previous analytical research on v-Na-Al glass by Robertshaw *et al*. [4] and Dussubieux and Kusimba [8], which was mostly based on major and minor elements or by comparing their results with a relatively limited number of glass assemblages.

What we have done here is to define compositional groups by using Zr, Ti, Ba, Cr and La associated with sand sources as discriminators (Table 4). Published compositional results for comparable glasses with no trace element results have been excluded due to the difficulty of dividing groups using major and minor elements only.

**Table 4:** The means and standard deviations of different high alumina-plant ash glass types in Africa and southeast Asia dating to between the 9^th^ and 16^th^ centuries AD.

	Type A				Type B	Type C	Type D
	Malindi glass vessel	Mamrbui glass beads	Mapungubwe Oblate series	Madagascar	Zimbabwe series	Mtwapa	Pengalan Bujang
Date	15^th^– 16^th^ century	15^th^– 16^th^ century	13^th^– 14^th^ century	13^th^– 14^th^ century	14^th^– 15^th^ century	10^th^– 17^th^ centuries	12^th^– 13^th^ century
NaO	15.86% ± 0.93%	16.08% ± 1.11%	12.40% ± 2.09%	13.01% ± 1.84%	14.56% ± 2.89%	18.78% ± 1.27%	17.68% ± 1.02
MgO	4.41% ± 0.30%	4.33% ± 0.59%	5.28% ± 1.84%	3.92% ± 0.50%	4.01% ± 0.74%	5.28% ± 0.44%	4.54% ± 0.72%
Al2O3	5.67% ± 0.39%	5.55% ± 0.41%	7.18% ± 1.43%	4.96% ± 1.20%	6.51% ± 1.14%	5.73% ± 0.68%	4.95% ± 0.58%
K2O	3.10% ± 0.28%	3.15% ± 0.55%	3.30% ± 0.58%	3.12% ± 0.77%	3.54% ± 0.60%	2.72% ± 0.29%	2.53% ± 0.23%
CaO	5.48% ± 1.02%	5.50% ± 0.87%	6.13% ± 1.73%	4.29% ± 0.72%	6.54% ± 1.30%	5.08% ± 0.47%	4.02% ± 0.43%
Ti	2081 ± 430	2084 ± 418	2556 ± 688	2020 ± 723	2370 ± 407	1532 ± 270	1136 ± 180
Sr	377 ± 56	361 ± 46	489 ± 120	352 ± 125	487 ± 104	400 ± 92	283 ± 35
Zr	69 ± 10	69 ± 12	119 ± 25	110 ± 52	200 ± 38	112 ± 12	94 ± 14
Ba	417 ± 21	407 ± 30	486 ± 126	353 ± 191	635 ± 174	679 ± 816	179 ± 69
1000Zr/Ti	34 ± 3	34 ± 3	48 ± 15	56 ± 24	86 ± 16	75 ± 10	84 ± 15
Cr/La	3.72 ± 0.54	3.53 ± 0.65	3.88 ± 1.51	3.99 ± 2.71	3.63 ± 1.28	2.00 ± 0.32	1.57 ± 0.45

Only selected major oxides and trace elements associated with sand and plant ash sources are presented here.

(1) Type A–consists of 15^th^– 16^th^ century AD Malindi glass vessels and Mambrui glass beads, glass beads from 13^th^– 14^th^ century AD Madagascar [3] and the 13^th^– 14^th^ century AD Mapungubwe Oblate series [4]. This group is characterised by elevated levels of Ti and Ba and relatively high Cr/La ratios, but with low 1000Zr/Ti ratios. It is likely that these glasses were manufactured in the same glassmaking tradition and appeared in the 13^th^– 14^th^ centuries AD in southern and eastern Africa. But by the 15^th^– 16^th^ centuries AD, a subgroup of Type A was found in Malindi and Mambrui and seems to have replaced the earlier subgroup of Type A glass.

Although their overall characteristics remain the same, we have noticed some glass compositional differences between the Malindi and Mambrui glass on the one hand, and the earlier the Mapungubwe Oblate series and Madagascar beads on the other hand. It has been noted above that different plant species/genera and/or plants from different geological locations might have been used to produce the Malindi and Mambrui glass assemblages, based on the differences in the concentrations of NaO, MgO, Li and CaO. Moreover, the somewhat lower Zr concentrations (Fig 10) and 1000Zr/Ti ratios (Fig 9) probably indicates local variations in the mineralogical characteristics of the sands used and suggests that they were made in different workshops in different geological locations from the Madagascan glass beads and the beads constituting the Mapungubwe Oblate series. Therefore, the authors believe the Malindi and Mambrui glasses represent a subgroup of Type A.

(2) Type B– 14^th^– 15^th^ century AD Zimbabwe series: Despite the use of a compositionally similar plant ash to make especially the Malindi and Mambrui glass, Type B has the highest concentrations of Zr and Ba out of all other groups and a relatively high ratio of Cr/La. A distinctive sand source must have been used to produce the glass in this group.

(3) Type C– 10^th^– 17^th^ centuries AD Mtwapa glass vessels: this group has lower levels of Ba and Ti than Types A and B and lower concentrations of Zr than Type B. Mtwapa glass vessels also have lower Cr/La ratios than Types A and B indicating that sand sources with different mineralogical signatures were used to produce Type C. The higher levels of Na_2_O in Type C shows that a distinctive plant genera/species was used. Current evidence suggests this type of v-Na-Al glass was only used to make glass vessels and can only be found in Mtwapa in Kenya [8].

(4) Type D– 12^th^– 13^th^ century AD Pengalan Bujang: similar to Type C, this group has lower concentrations of Ba and Ti than Types A and B as well as lower concentrations of Zr than Type B. What distinguishes Type D from Type C is the concentrations of CaO, MgO, Ti, Sr and the Cr/La ratios. Type D has lower concentrations of CaO, MgO, Ti and Sr than Type C, and higher Cr/La ratios. This shows that different plant ashes and sand sources were used to make it. This type of v-Na-Al glass has only been found in Malaysia and does not seem to have been used in Africa. Only glass vessels were made with this glass type [9].

#### The provenance of v-Na-Al glasses

While we can identify different compositional groups of v-Na-Al glass, the provenance of v-Na-Al glass remains elusive. The lack of archaeological evidence for primary glass workshop(s) that fused plant ash and sand to make v-Na-Al glass means we are still unable to provenance individual compositional group of v-Na-Al glass. There is no conclusive evidence to suggest that v-Na-Al glass was manufactured in eastern and southern Africa; in Shanga, eastern Africa, only evidence for glass bead making was found [40]. It is assumed that these glass artefacts were manufactured outside Africa and imported from regions that were known to have produced v-Na-Al glass. Possible sources are Central Asia, such as Kuva and Akhsiket in eastern Uzbekistan, suggested by Dussubieux and Kusimba [8], Then-Obluska and Dussubieux [41] and Carter *et al*. [42]; India and southeast Asia have also previously been suggested as the possible production regions for v-Na-Al glass [4, 8, 24, 29, 43]. However, the lack of archaeological and archaeometric data on sub-Saharan African glass means that we cannot fully rule out the possibility that v-Na-Al glass could have been made and/or worked into glass vessels and beads in Africa, although this seems unlikely.

Recent analytical research on glass from Termez in southern Uzbekistan (Henderson unpublished), Kuva and Akhsiket (a primary production glass centre) in eastern Uzbekistan [29, 44] and Ghazni in Afghanistan [36] may provide some clues about the provenance of v-Na-Al glass. The Uzbek and Afghan glasses are characterised by elevated levels of Al_2_O_3_, high MgO as well as relatively low ratios of Cr/La and 1000Zr/Ti [29, 35, 36]. They are different from Middle Eastern plant ash glass which generally has higher Cr/La and lower levels of Al_2_O_3_ [10].

Looking at the major and minor elements, some of the Kuva and Akhsiket glasses and a small number of Ghazni glass have high concentrations of Al_2_O_3_ similar to v-Na-Al glasses from Africa and southeast Asia [29, 36]. However, it should also be noted that some Central Asian including Ghazni glasses have Al_2_O_3_ below 4% [29, 44, Henderson pers. comm.] and may reflect different production zones within it. It is also noted that some of these high Al_2_O_3_ Ghazni, Kuva and Akhsiket glasses have K_2_O below 4%, similar to v-Na-Al glass (Table 4). Henderson *et al*. [7] suggest the plants used to make the Uzbek glasses grew on a contrasting geology to those used to make other Central Asian glasses with high K_2_O.

The Kuva and Ghazni glasses have similarly high MgO/CaO ratios to Type A, while most of the Akhsiket glass has lower levels of MgO than the majority of the v-Na-Al glass [29, 36]. However, the Na_2_O concentrations of the Kuva and Ghazni glass are significantly higher than most Type A glasses comparable to Types C and D, while most of the Akhsiket glass has similar concentrations of Na_2_O to Type A glasses [29, 36].

Further, trace element analysis reveals similarities between the Central Asian, including Ghazni and v-Na-Al glasses. It can be seen in Fig 9 that the majority of the Ghazni glass has especially low ratios of 1000Zr/Ti and Cr/La similar to v-Na-Al glass. The Termez and Ghazni glasses fall within the area of the Mtwapa and Pengalan Bujang glass (Fig 9). The Ghazni glass also has rather low levels of Ba (Fig 8) and Sr. The levels of B in Ghazni glass, which is associated with the alkali source, are also relatively high (>190ppm) [36].

Fiorentino *et al*. [36] suggest the glass vessels and bracelets from Ghazni in Afghanistan belong to the so-called Mesopotamian type 1 compositional group, which includes glasses from Veh Ardasir, Samarra and Ctesiphon in Iraq and Nishapur in Iran [36].

However, while most v-Na-Al, Central Asian and the Ghazni glasses may have similar MgO/CaO ratios and Na_2_O level to glass assemblages from the area consisting of modern day Iran and Iraq, Cs/K and Li/K ratios in v-Na-Al glasses and some Ghazni glasses have significantly lower Li/K ratios than Nishapur and Samarra glasses [10]. Although low Cs/K and Li/K ratios can be found in glass from the Levantine region and Egypt such as some 12^th^ - 14^th^ century AD glass vessels from Damascus and Beirut [10], some Central Asian glasses contain distinctly lower levels.

Because Levantine and Egyptian glasses have low Al_2_O_3_ levels, it is very unlikely that v-Na-Al, Central Asian and the Ghazni glasses were made in the region, or in the Mesopotamian region. Moreover, v-Na-Al glass and the Ghazni glass contain higher B levels than found in Mesopotamian and Levantine glasses [10, 36].

Furthermore, v-Na-Al glass, Central Asian glass from Ghazni are distinguishable from Nishapur and Samarra glasses based on the ratios of 1000Zr/Ti and Cr/La. Nishapur and Samarra glasses generally have higher 1000Zr/Ti and Cr/La ratios than v-Na-Al glass, Central Asian glass and the Ghazni glass [10]. Low Zr/Ti ratios appears to be a distinctive characteristic of Ghazni glasses; glasses from Samarra, Nishapur, Damascus and Cairo all have higher values. Low 1000Zr/Ti and Cr/La ratios are found in 12^th^– 14^th^ century AD Beirut colorless vessel glasses (Beirut samples 54 and 55) [10]. Both are distinctive because they contain more than 4% K_2_O (4.04% and 4.1% respectively) which in any case suggests a Central Asian origin [45]. While these 2 glasses have similarly low Cr/La ratios of c. 2, their 1000Zr/Ti values are higher than the Malindi and Mambrai glasses at c. 50 and therefore similar to Ghazni glasses. Moreover, ^143^Nd/^144^Nd and ^87^Sr/^86^Sr signatures for these 2 glasses are distinctive [45].

Therefore, our analysis shows that the Ghazni glass is compositionally similar to v-Na-Al glass and Central Asian glass and it is unlikely to have been made in Iraq or Iran as was suggested by Fiorentino *et al*. [36] or in the Levantine and Egyptian regions. Trace element analyses suggests v-Na-Al and the Ghazni glass came from Central Asia. The Ghazni glass may well belong to a type of v-Na-Al glass circulating in Central Asia including Afghanistan.

**Fig 11:**
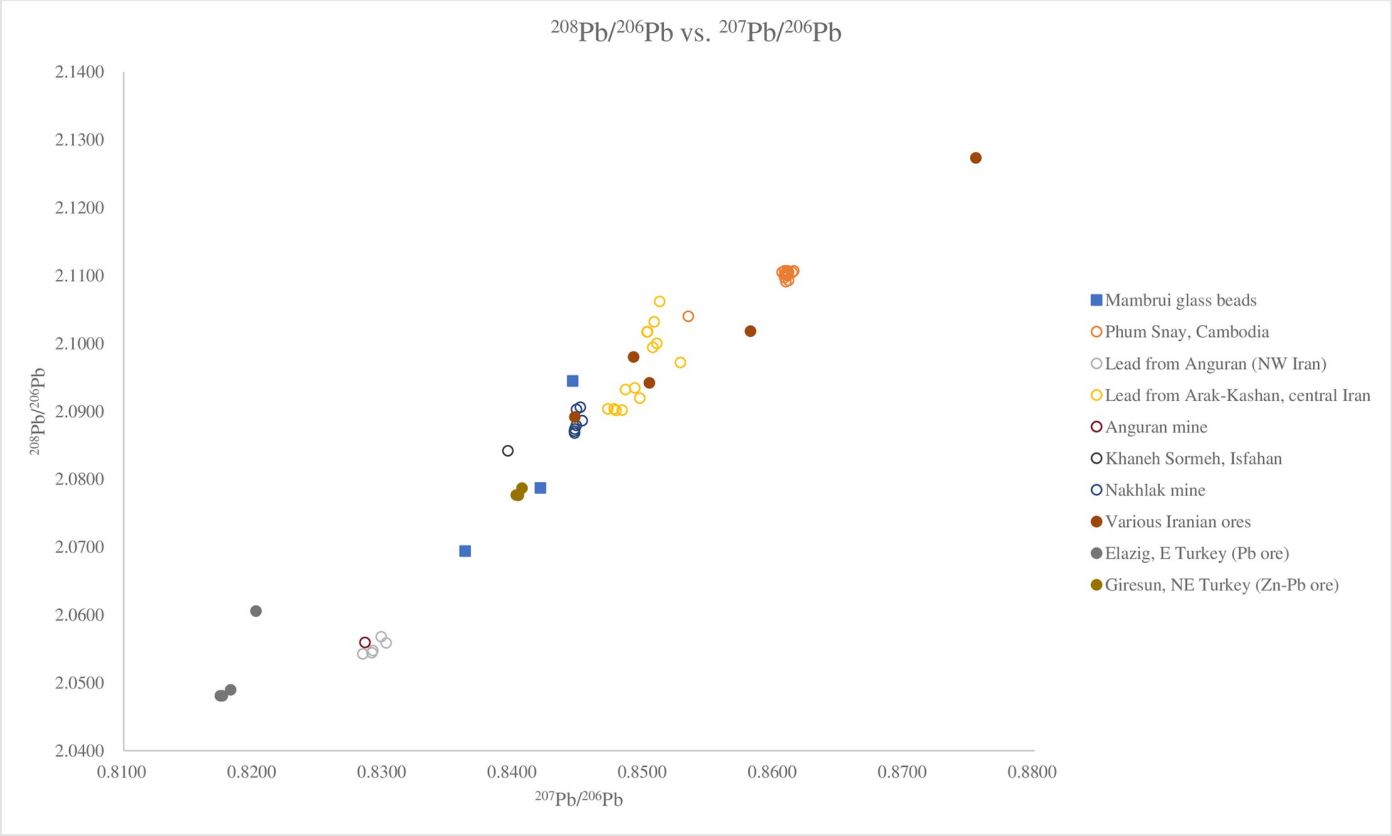
Lead isotope ratios ^208^Pb/^206^Pb and ^207^Pb/^206^Pb of glass beads from Mambrui, ores from Iran and Afghanistan and artefacts from Afghanistan, India, Indonesia, Cambodia and Iran. Data are from Phum Snay [48]; Lead from Anguran and Arak-Kashan, Anguran mine, Khaneh Sormeh, Nakhlak mine and various Iranian ores [46]; Farinjal ores and glazes and a sherd of Ghazni glaze [47]; Elazig and Giresun [49]. It is noted that the Mambrui glass beads with high lead contents have similar lead isotope ratios to lead ores from Khaneh Sormeh, Nakhlak mines and Kouchke in Iran, and a tin-lead ore from Giresun in northeastern Turkey.

**Fig 12:**
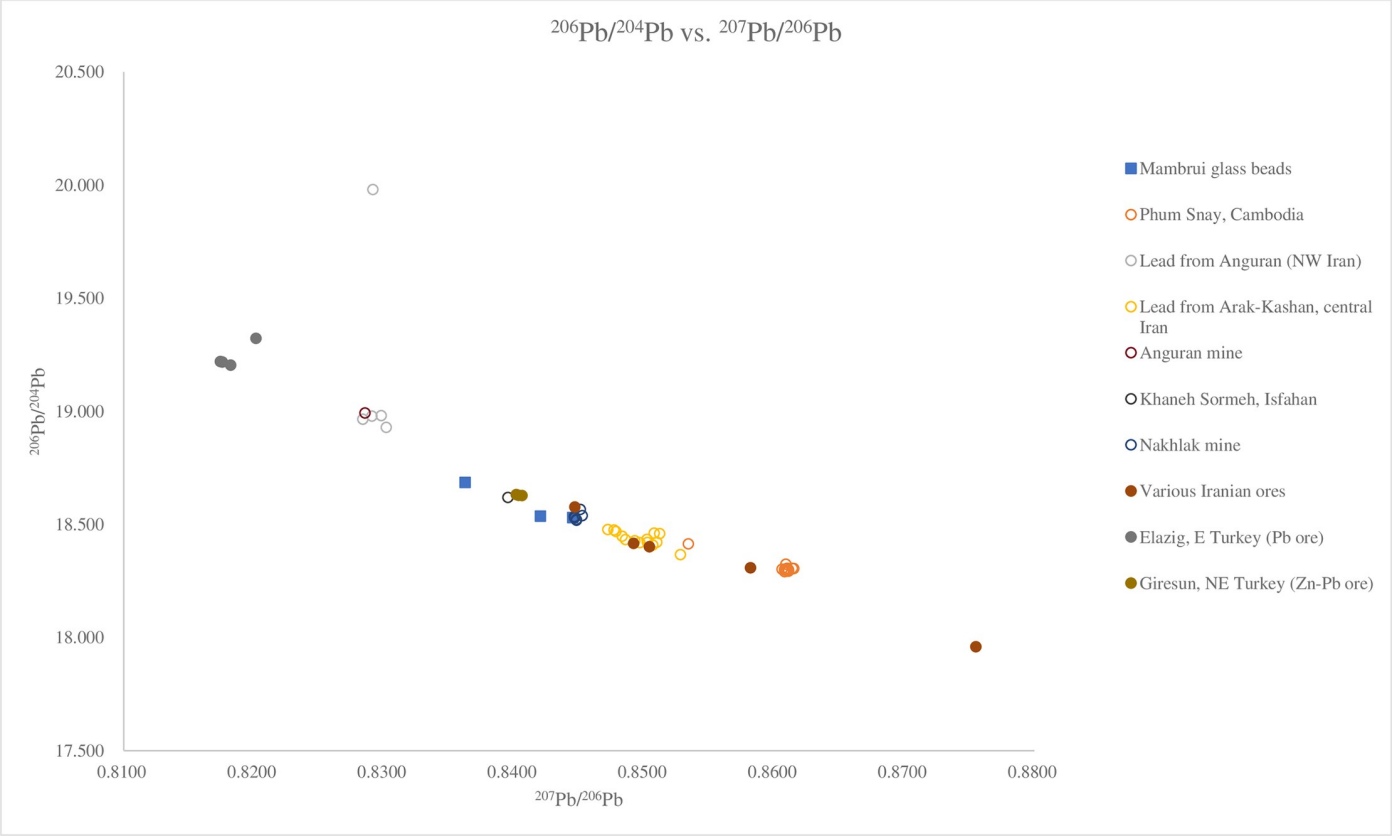
Lead isotope ratios ^206^Pb/^204^Pb and ^207^Pb/^206^Pb of glass beads from Mambrui, ores from Iran and Afghanistan and artefacts from Afghanistan, India, Indonesia, Cambodia and Iran. Data are from Phum Snay [48]; Lead from Anguran and Arak-Kashan, Anguran mine, Khaneh Sormeh, Nakhlak mine and various Iranian ores [46]; Farinjal ores and glazes and a sherd of Ghazni glaze [47]; Elazig and Giresun [49]. It is noted that the Mambrui glass beads with high lead contents have similar lead isotope ratios to lead ores from Khaneh Sormeh, Nakhlak mines and Kouchke in Iran, and a tin-lead ore from Giresun in northeastern Turkey.

Lead isotope analysis suggests opaque and opaque yellow glass from Mambrui might have been produced with lead-tin opacifiers and colourants originating from Turkey and the Middle East such as Iran. Glass beads, B41, B45 and B50, fall within a range of 2.0694–2.0945 for ^208^Pb/^206^Pb and within a range of 0.8362–0.8445 for ^207^Pb/^206^Pb, which is similar to a tin-lead ore from Giresun in northeastern Turkey, a lead ore found in Khaneh Sormeh in Isfahan, Iran, Nakhlak mine in Iran and two glazes from Farinjal in Afghanistan (Fig 11) [46, 47]. The ^206^Pb/^204^Pb ratios of these four beads also suggest the origin of the lead contents in the glass might have come from these regions which share similar lead isotope ratios (Fig 12) [46].

At present, we can rule out the possibility that the high tin and lead glass beads were produced with lead sources from southeast Asia such as the Song Toh lead mine in Thailand [48] (Figs 11 and 12), which has different lead isotope ratios from the Mambrui glass beads.

However, a disadvantage of lead isotope analysis is that it is difficult to separate between regions with similar lead isotopic ratios, thus making it difficult to rule out regions with similar isotopic ratios. Moreover, we cannot rule out the possibility that tin-based opacified glass of different origins was recycled and the isotopic signatures of the opacifiers have been distorted. Therefore, at this stage, we can only suggest the source for the lead used in the beads most likely came from regions in Asia Minor and the Middle East, such as Turkey and Iran and can safely rule out regions in southeast Asia and south Asia.

The authors intend to analyse the Malindi and Mmabrui glass further using Sr and Nd isotope in order to investigate its provenance in more detail [50]. In recent years, radiogenic isotopes (e.g. Sr and Nd isotopes) are proven to be effective to in provenancing ancient glass and sources of sand raw materials [51–53].

## Conclusion

Based on the EPMA and LA-ICP-MS analyses, the glass analysed here from Malindi and Mambrui in Kenya can be classified as the plant ash high alumina glass type. This type of glass is mostly found in Africa and southeast Asia in the 9^th^– 16^th^ centuries AD. Combining our results, especially for Cr, La, Ti, Zr, Ba, Cs and Li, with published research on v-Na-Al glass, we have reviewed the existing compositional characteristics of v-Na-Al glass and are able to identify at least 4 types, of which the Kenyan glass belongs to Type A. The similarity between Central Asian glass from Uzbekistan, v-Na-Al and the Ghazni glass in Afghanistan indicates strongly that v-Na-Al glass of type A was manufactured in Central Asia. Lead isotope analysis further suggests lead-tin opacifiers and colourants came from ores in Turkey and/or Middle East such as Iran.

Our results also reveal a complex glass trading network across Central Asia, south Asia and Africa in the 9^th^– 16^th^ centuries AD. While the glass vessels (along with their contents) could have been traded directly from Central Asia, where plant ash glass vessels are known to have been made, to Africa to sites such as Malindi and Mtwapa, there is very little evidence that Central Asian glass was traded directly to Africa. We therefore believe that Central Asian glass could have reached Africa via India, where a thriving glass industry existed in the 9^th^– 16^th^ centuries AD.

There is ample historical and archaeological evidence to support this trading network. Cultural and trading relationships between Central Asia and India are well attested in historical and archaeological records. For instance, the conquest of India by people from Central Asia in the 10^th^– 13^th^ centuries AD led to the growth of caravan trade between India and the Muslim East. Coins of the Delhi Sultanate are found in Central Asia [54, 55]. By the 13^th^ century AD, Delhi became a melting pot of Indo-Muslim culture in India. Scientists and poets from Otyrar, Samarkand, Bukhara and Balh found a ‘new Motherland in India and enriched its culture by their work and increased its glory’ [55].

Indian-Central Asian relations continued into the 16^th^ century AD. The establishment of the Mughal Empire in northern India encouraged and intensified cultural and trading contacts with Central Asia. Indian merchants from northern India started trading in the north to Afghanistan, Iran, Central Asia and Kazakhstan [55]. Therefore, it is not impossible that v-Na-Al glass from Central Asia was exported to India, where glass bead making was most renowned in the 9^th^– 16^th^ centuries AD. It was from India where Central Asian v-Na-Al glass and (recycled) opacifiers and colourants from Asia Minor and/or Middle East such as Iran were made into beads and subsequently exported to Africa. But further archaeological and scientific investigations on v-Na-Al glass are needed to provide more evidence to support this hypothesis.

Indian glass beads were prized by locals in Africa from the 9^th^– 16^th^ centuries AD. Portuguese records indicate that locals in Africa were unwilling to accept European-made beads and demanded beads that were made in India [56]. Glass beads, such as the East Coast Indo-Pacific series, are said to have been manufactured in northern India and traded to east African coastal cities such as Shanga in the 10^th^– 13^th^ centuries AD [57]. Glass beads from India continued to be exported to Africa before European beads took over the market in the 17^th^ century AD. Therefore, we believe that Central Asian glass could have been traded directly to India where Central Asian glass was manufactured into glass beads, which were then traded to Africa. But so far there are no v-Na-Al glass has ever been found in India, further archaeological and scientific researches on Indian glass and v-Na-Al glass are needed to further support this hypothesis and to ascertain whether v-Na-Al glass was used to make glass beads in India.

## Supporting information

S1 Table The results of the analysis of the Corning Glass Standard B (expressed in wt%). The known composition of the Corning Glass Standard B is from Brill (1999). Bdl = below detection limit.
(XLSX)

S2 Table The results of the analysis of the NIST SRM 610 and 612, BHOV-2G, BCR-2G and BIR-1G standards (expressed in wt% and ppm). The known composition of the standards are from Jochum et al. [1].
(XLSX)

S3 Table The results of the analysis of SRM981. The known ratios of SRM981 is from Cui *et al*. [21].
(XLSX)

S4 Table Reduced compositions of the Malindi glass vessels and Mambrui glass beads from Kenya. For the glass vessels, we have used EPMA results for major elements (Na2O*, MgO*, Al2O3*, SiO2*, K2O* and CaO*) and LA-ICP-MS results for minor element FeO*. LA-ICP-MS results were used for the glass beads.
(XLSX)

S5 Table EPMA and LA-ICP-MS results for glass vessels from Malindi and glass beads from Mambrui in Kenya. Results are presented in weight percent oxide/element and ppm/element. EPMA results for the major and some minor element oxides (Na_2_O, SiO_2_, CaO, MgO, K_2_O and Al_2_O_3_) and the LA-ICP-MS results for the minor and trace elements. LA-ICP-MS results were used for the glass beads.
(XLSX)

S6 Table Lead isotope analysis results for glass beads from Mambrui, Kenya. (XLSX)

## References

[1] Wood M, Dussubieux L, Robertshaw P. The glass of Chibuene, Mozambique: new insights into early Indian Ocean trade. South African Archaeological Bulletin 2012; 67: 59–74.

[2] Wood M, Panighello S, Orsega EF, Robertshaw P, van Elteren JT, Crowther A, et al Zanzibar and Indian Ocean trade in the first millennium CE: the glass bead evidence. Archaeological and Anthropological Sciences 2017; 9: 879–901.

[3] Robertshaw P, Rasoarifetra B, Wood M, Melchiorre E, Popelka-Filcoff RS, Glascock MD. Chemical analysis of glass beads from Madagascar. J.Afr. Arch. 2006; 4: 91–109.

[4] Robertshaw P, Wood M, Melchiorre E, Popelka-Filcoff RS, Glascock M. Southern African glass beads: chemistry, glass sources and patterns of trade. Journal of Archaeological Science 2010; 37: 1898–1912.

[5] Dussubieux L, Kusimba CM, Gogte V, Kusimba SB, Gratuze B, Oka R. The trading of ancient glass beads: new analytical data from south Asian and east African soda-alumina glass beads. Archaeometry 2008; 50: 797–821.

[6] Dussubieux L, Gratuze B, Blet-Lemarquand M. Mineral soda alumina glass: occurrence and meaning. Journal of Archaeological Science 2010; 37: 1646–1655.

[7] Henderson J, An J, Ma H. The archaeometry and archaeology of ancient Chinese glass: a review. Archaeometry 2018; 60: 88–104.

[8] Dussubieux L, Kusimba CM. Glass Vessels in Sub-Saharan Africa: Compositional Study of Some Samples from Kenya. In: Liritzis I, Stevenson C editors. The Dating and Provenance of Obsidian and Ancient Manufactured Glasses. Albuquerque: University of New Mexico Press; 2012 pp. 143–156.

[9] Dussubieux L, Allen J. Chemical Compositions of Glass Artifacts from Malaysia: New Data from the Sites of Kampung Pengkalan Bujang and Kampung Sungai Mas In: Perret D, Jaafar ZB editors. Ancient Glassware in Malaysia: The Pengkalan Bujang Collection. Kuala Lumpur: Department of Museums Malaysia; 2014 pp. 119–161.

[10] Henderson J, Chenery S, Faber E, Kröger J. The use of electron probe microanalysis and laser ablation-inductively coupled plasma-mass spectrometry for the investigation of 8th– 14th century plant ash glasses from the Middle East. Microchemical Journal 2016; 128: 134–152.

[11] Shortland A, Rogers N, Eremin K. Trace element discriminants between Egyptian and Mesopotamian late Bronze age glass. Journal of Archaeological Science 2007; 34: 781–789.

[12] Siu I. Cui JF, Henderson J, Ding Y, Qin DH. A study of 11^th^– 15^th^ centuries AD glass beads from Mambrui, Kenya: an archaeological and chemical approach. Journal of Archaeological Science: Reports. Forthcoming 2020.

[13] Ding Y. Archaeological research on Mambrui and Malindi in Kenya. Unpublished PhD thesis: Peking University; 2015.

[14] Qin DS, Ding Y. Mambrui and Malindi. In: Wynne-Jones S, LaViolette A. editors. The Swahili World. London and New York; Routledge; 2018 pp. 205–213.

[15] Henderson J. Electron probe microanalysis of mixed-alkali glasses. Archaeometry 1988; 30: 77–91.

[16] Meek A, Henderson J, Evans J. Isotope analysis of English forest glass from the Weald and Staffordshire. J. Anal. At. Spectrom. 2012; 27: 786–795.

[17] Liu Y, Hu Z, Gao S, et al In-situ analysis of major and trace elements of anhydrous minerals by LA-ICP-MS without applying an internal standard. Chemical Geology 2008; 257: 34–43.

[18] Pearce NJG, Perkins WT, Westgate JA, et al A compilation of new and published major and trace element data for NIST SRM 610 and NIST SRM 612 glass reference materials. Geostandards Newsletter 1997; 21: 115–144.

[19] Gratuze B. Glass characterization using laser ablation-inductively coupled plasma-mass spectrometry methods In: Dussubieux L, Gratuze B, Golitko M, editors. Recent advances in laser ablation ICP-MS for archaeology. Berlin: Springer; 2016 pp. 179–196.

[20] Cui JF, Lei Y, Jin ZB, Huang BL, Wu XH. Lead isotope analysis of Tang sancai pottery glazes from Gongyi Kiln, Henan province and Huangbao kiln, Shaanxi province. Archaeometry 2010; 52: 597–604.

[21] Cui JF, Wu XH, Huang BL. Chemical and lead isotope analysis of some lead-barium glass wares from the Warring States Period, unearthed from Chu tombs in Changde City, Hunan Province, China. Journal of Archaeological Science 2011; 38: 1671–1679.

[22] Henderson J. Ancient Glass: An interdisciplinary exploration. Cambridge: Cambridge University Press; 2013.

[23] Dussubieux L. Compositional Analysis of Ancient Glass Fragments from North Sumatra, Indonesia In: Perret D, Surachman H, editors. Histoire de Barus III: Regards Sur Une Place Marchande de l'océan Indien (XIIe-Milieu Du XVIIe S.). Paris: Association Archipel/EFEO; 2009 pp. 385–417.

[24] McKinnon EE, Brill RH. Chemical analyses of some glasses from Sumatra. Section 2 In: Bhardwaj HC, editor. Archaeometry of Glass. Proceedings of the Archaeometry Session of the XIV International Congress on Glass 1986 New Delhi India. Calcutta: India Ceramic Society; 1987 pp. 1–14.

[25] Pollard AM, Heron C. Archaeological chemistry 2^nd^ edition Cambridge: The Royal Society of Chemistry; 2008.

[26] Henderson J. The raw materials of early glass production. Oxford Journal of Archaeology 1985; 4: 267–291.

[27] Barber DJ, Freestone I, Moulding KM. Ancient copper red glasses: investigation and analysis by microbeam techniques In: Shortland AJ, Freestone IC, Rehren Th. editors. From mine to microscope. Oxford: Oxbow Books; 2009 pp. 115–127.

[28] Tamura T, Oga K. Archaeometrical investigation of natron glass excavated in Japan. Microchemical Journal 2015; 126: 7–17.

[29] Rehren T, Osório A, Anarbaev A. Some Notes on Early Islamic Glass in Eastern Uzbekistan In: Bettina Z, Hilgner A, editors. Glass along the Silk Road from 200 BC to AD 1000. Mainz: Verlag des Römisch-Germanischen Zentralmuseums; 2010 pp. 93–103.

[30] Sode T, Gratuze B, Lankton J. Red and orange high-alumina glass beads in 7^th^ and 8^th^ century Scandinavia: evidence for long distance trade and local fabrication In: Wolf S, de Pury-Gysel A, editors. Annales du 20^e^ congrès de l’association internationale pour l’histoire du verre, (Fribourg /Romont 7–11 Septembre 2015). Romont: Verlag Marie Leidorf GmbH; 2017 pp. 326–333.

[31] Brill RH. Chemical analyses of some glass fragments from Nishapur in the Corning Museum of Glass In: Kröger J, editor. Nishapur. Glass of the Early Islamic Period. New York: The Metropolitan Museum of Art; 1995 pp. 211–233.

[32] Freestone IC. Composition and Affinities of Glass from the Furnaces on the Island site, Tyre. Journal of Glass Studies 2002; 44: 67–77.

[33] Freestone IC, Gorin-Rosen Y, Hughes MJ. Primary glass from Israel and the production of glass in late antiquity and the early Islamic period In: Nenna M-D, editor La route du verre: ateliers primaires et secondaires du second millenaire av. J.-C. au Moyen age. Lyon: Maison de l’Orient Mediterraneen-Jean Pouilloux; 2000 pp. 65–84.

[34] Henderson J, McLoughlin S, McPhail D. Radical changes in Islamic glass technology: evidence for conservatism and experimentation with new glass recipes from early and middle Islamic Raqqa, Syria. Archaeometry 2004; 46: 439–68.

[35] Schibille N, Meek A, Wypyski MT, Kröger J, Rosser-Owen M, Wade Haddon R. The glass walls of Samarra (Iraq): ninth-century Abbasid glass production and imports. PLoS ONE 2018; 13: e0201749 10.1371/journal.pone.0201749 30133468 PMC6104971

[36] Fiorentino S, Venezia B, Schibille N, Vandini M. Streams across the Silk Roads? The case of Islamic glass from Ghazni. Journal of Archaeological Science: Reports 2019; 25: 153–170.

[37] Barkoudah Y, Henderson J. Plant ashes from Syria and the manufacture of ancient glass: ethnographic and scientific aspects. Journal of Glass Studies 2006; 48: 297–321.

[38] Shen JY, Henderson J, Evans J, Chenery S, Zhao FY. A study of the glazing techniques and provenances of Tang Sancai glazes using elemental and lead isotope analyses. Archaeometry 2019; 61: 358–373.

[39] Oikonomou A, Henderson J, Gnade M, Chenery S, Zacharias N. An archaeometric study of Hellenistic glass vessels: evidence for multiple sources. Archaeological and Anthropological Sciences 2018; 10: 97–110.

[40] Horton M. Shanga: the archaeology of a Muslim trading community on the coast of east Africa. London: The British Institute in Eastern Africa; 1996.

[41] Then-Obluska J, Dussubieux L. Glass bead trade in the early Roman and Mamluk Quseir ports–a view from the Oriental Institute Museum assemblage. Archaeological Research in Asia 2016; 6: 81–103.

[42] Carter A, Dussubieux L, Polkinghorne M, Pottier C. Glass artifacts at Angkor: evidence for exchange. Archaeological and Anthropological Sciences 2019; 11: 1013–1027.

[43] Brill RH. Chemical analyses of some early Indian glasses In: Bhardwaj HC, editor. Archaeometry of glass: proceedings of the archaeometry session of the XIV international congress on glass, 1986, New Delhi, India. Calcutta: Indian Ceramic Society; 1987 pp. 1–25.

[44] Liu S, Li QH, Gan F, Zhang P, Lankton JW. Silk road glass in Xinjiang, China: chemical compositional analysis and interpretation using a high-resolution portable XRF spectrometer. Journal of Archaeological Science 2012; 39: 2128–2142.

[45] Henderson J, Ma H, Evans J. Glass production for the Silk Road? Provenance and trade of Islamic glasses using isotopic and chemical analyses in a geological context. Journal of Archaeological Science [Preprint]. 2020 [cited 2020 May 18]: [12 p.]. Available from: https://doi.org/10/1016/j.jas.2020.105164

[46] Stos-Gale S. Lead-isotope analyses of glass, glazes and some metal artifacts In: Bass GF, Matthews SD, Steffy JR, van Doorninck Jr. FH, editors. Serce Limani: an eleventh-century shipwreck Volume I Texas: Texas A&M University Press; 2004 pp. 453–467.

[47] Brill RH, Felker-Dennis C, Shirahata H, Joel EC. Lead isotope analyses of some Chinese and Central Asian pigments In: Agnew N, editor. Conservation of ancient sites on the silk road–proceedings of an international conference on the conservation of grotto sites (Dunhuang, October 1993). Los Angeles: Getty Conservation Institute; 1997 pp. 369–378.

[48] Hirao Y, Ro J-H. Chemical composition and lead isotope ratios of bronze artifacts excavated in Cambodia and Thailand In: Yasuda Y, editor. Water Civilization: from Yangtze to Khmer civilizations. Tokyo: Springer; 2013 pp. 247–312.

[49] Turkish ores [Internet]. OXALID: Oxford archaeological lead isotope database from the isotrace laboratory. C 2009 –[cited 2020 May 10]. Available from: http://oxalid.arch.ox.ac.uk/Turkey/Turkey.html

[50] Siu I, Cui JF, Henderson J, Ding Y, Qin DH. The provenance of v-Na-Al glass from Malindi and Mambrui, Kenya: a strontium and neodymium isotope analysis. In preparation, 2020.

[51] Freestone IC, Leslie KA, Thirlwall M, Gorin-Rosen Y. Strontium isotopes in the investigation of early glass production: Byzantine and early Islamic glass from the Near East. Archaeometry 2003; 45, 19–32.

[52] Henderson J, Evans J, Barkoudah Y. The roots of provenance: glass, plants and isotopes in the Islamic Middle East. Antiquity, 2009; 83, 414–429.

[53] Brems D, Ganio M, Degryse P. The Sr-Nd isotopic fingerprint of sand raw materials In: Degryse P editor. Glass making in the Greco-Roman world. Leuven: Leuven University Press; 2014 pp. 51–68.

[54] Kumar BB. India and Central Asia: links and interactions In Roy JN, Kumar BB, editors. India and Central Asia. Classical to contemporary periods. New Delhi: Concept Publishing Company; 2007, 3–33.

[55] Abuseitova MKh. Historical and cultural relations between Kazhakhstan, Central Asia and India from ancient times to the beginning of the 20^th^ century In Roy JN, Kumar BB, editors. India and Central Asia. Classical to contemporary periods. New Delhi: Concept Publishing Company; 2007, 43–56.

[56] Wood M. Glass beads and Indian Ocean trade In Wynne-Jones S, LaViolette A, editors. The Swahili world. London and New York: Routledge; 2018 pp. 458–471.

[57] Wood M. Interconnections–glass beads and trade in southern and eastern Africa and the Indian Ocean– 7^th^– 16^th^ centuries AD [dissertation]. Uppsala: Uppsala Universitet; 2012.

